# Biodiversity data and new species descriptions of polychaetes from offshore waters of the Falkland Islands, an area undergoing hydrocarbon exploration

**DOI:** 10.3897/zookeys.938.49349

**Published:** 2020-06-03

**Authors:** Lenka Neal, Gordon L. J. Paterson, David Blockley, Ben Scott, Emma Sherlock, Cate Huque, Adrian G. Glover

**Affiliations:** 1 Natural History Museum, London, UK Natural History Museum, London London United Kingdom; 2 Greenland Institute of Natural Resources, Nuuk, Greenland Greenland Institute of Natural Resources Nuuk Greenland

**Keywords:** Annelida, biodiversity, DarwinCore, deep sea, Environmental Impact Assessments, Falkland Islands, hydrocarbon exploration, marine, oceans, taxonomic novelty

## Abstract

Benthic environmental impact assessments and monitoring programs accompanying offshore hydrocarbon industry activities result in large collections of benthic organisms. Such collections offer great potential for systematics, biodiversity and biogeography research, but these opportunities are only rarely realised. In recent decades, the hydrocarbon industry has started exploration activities in offshore waters off the Falkland Islands. A large collection of ca. 25,000 polychaete (Annelida) specimens, representing some 233 morphological species was processed at the Natural History Museum, London. Taxonomic assessment led to recognition of many polychaete species that are new to science. The existing taxonomic literature for the region is outdated and many species in existing literature are likely misidentifications. Initially, an online taxonomic guide (http://falklands.myspecies.info) was created, to provide a single taxonomic source for 191 polychaete species to standardise identification across different environmental contractors working in Falkland Islands. Here, this effort is continued to make data available for 18,015 specimens through publication of raw biodiversity data, checklist with links to online taxonomic information and formal descriptions of five new species. New species were chosen across different families to highlight the taxonomic novelty of this area: *Apistobranchus jasoni* Neal & Paterson, **sp. nov.** (Apistobranchidae), *Leitoscoloplos olei* Neal & Paterson, **sp. nov.** (Orbiniidae), *Prosphaerosyllis modinouae* Neal & Paterson, **sp. nov.** (Syllidae) and *Aphelochaeta falklandica* Paterson & Neal, **sp. nov.**, and *Dodecaceria saeria* Paterson & Neal, **sp. nov.** (both Cirratulidae). The potential of the Falkland Islands material to provide up to date informationfor known species described in the literature is also highlighted by publishing images and redescription of *Harmothoe anderssoni* Bergström, 1916 and *Aphelochaeta longisetosa* (Hartmann-Schröder, 1965). Biodiversity and abundance data are made available through a DarwinCore database, including material collected from 83 stations at Sea Lion developmental oil field in North Falklands Basin and voucher specimens’ data collected from exploratory oil wells in East Falklands Basin.

## Introduction

Benthic Environmental Impact Assessments (BEIAs) generate large amount of data, which can provide a comprehensive characterisation of benthic habitats and associated fauna. However, several conditions need to be met for this potential to be realised. Most importantly the samples have to be collected in standardised way; the identification of organisms needs to be carried out in a consistent manner and the data should be made available in the public domain. Lack of this consistency can make data incomparable in a broader context and prevent iterative improvement of species identifications and concepts. While in well-known areas, such as North Sea this may be less of a problem, the case is magnified once the surveys move to previously unsampled regions and/or depths (see e.g., Brett 2001; Glover et al. 2015; Dahlgren et al. 2016). The current process used for samples collected as part of BEIAs from deep sea (areas below 200 m depth) is disjointed, with samples scattered across many different contractors and sub-contractors, and mostly lacking a standardised taxonomy.

In recent decades, the potential for the development of a substantial offshore hydrocarbons industry within the Falkland Islands was realised (Blockley and Tierney 2017), with several benthic surveys carried out in 2008, 2009, 2011, and 2012. These surveys involved the identification and vouchering of large collections of benthic invertebrates from a poorly studied region of our planet. In this paper, we attempt to improve the data availability situation for offshore contracted work by making our species descriptions, raw data and vouchers available for future study as funded by the South Atlantic Environmental Research Institute (SAERI).

Marine benthic fauna from the Falklands area has been considered part of the Magellanic province, but links have also been drawn to the Southern Ocean and sub-Antarctic fauna (e.g., Arntz 1999). Available (and therefore used) taxonomicmonographs of polychaetes for these regions (Hartmann-Schröder 1962, 1965; Hartman 1966, 1967, 1978; Hartmann-Schröder and Rosenfeldt 1988, 1989, 1990, 1992), however, need updating. Recent taxonomic efforts, which concentrated on the polychaete family Ampharetidae (Schüller and Jirkov 2013) summed up the problem as follows: “The Southern Ocean and also Patagonian waters represent an interesting geographical region, as many [polychaete] species recorded from these areas were originally described from the Northern hemisphere. These records from Southern waters may possibly be the result of misidentification and give some substance for the discovery of new species from already well-defined genera”. For example, during ANDEEP (ANtarctic benthic DEEP-sea biodiversity: colonisation history and recent community patterns) expeditions to the deep Weddell Sea (Brandt et al. 2004) over 450 ampharetid specimens belonging to over 20 species (many undescribed) were found (Schüller et al. 2009; Schüller and Jirkov 2013). Similarly, recent BIOPEARL (Biodiversity, Phylogeny, Evolution and Adaptive Radiation of Life in Antarctica) expeditions to Scotia and, particularly, Amundsen Sea, revealed many species new to science for isopods, polychaetes, molluscs and tanaids (Kaiser et al. 2009; Neal et al. 2012, 2017; Moreau et al. 2013; Pabis et al. 2015).

The problem of the lack of standardised taxonomy between contractors, highlighted in the “Gap Analysis” carried out by SAERI (Blockley and Tierney 2017), was one of the priority issues in developing an understanding of ecosystem functioning. A potential solution was found initially by developing an on-line taxonomic guide (http://falklands.myspecies.info) to provide a standardised taxonomic resource, particularly for those species that are currently not formally described. An abundant and species-rich benthic group, the polychaete worms, was selected as a model taxon to demonstrate the value of a taxonomic resource based on a large collection (ca.25,000 specimens), collected as part of the BEIAs by the contractors and subsequently identified at NHM, London.

This paper is thus the first attempt at a partial, taxonomic synthesis for offshore waters of the Falkland Islands, based on currently available collections, and deals with description (formal or informal) of 191 polychaete taxa. Five new species from four families are formally described here to highlight the potential for discovery of species new to science in this material: *Apistobranchus jasoni* sp. nov. (Apistobranchidae), *Leitoscoloplos olei* sp. nov. (Orbiniidae), *Prosphaerosyllis modinouae* sp. nov. (Syllidae), *Aphelochaeta falklandica* sp. nov. and *Dodecaceria saeria* sp. nov. (both Cirratulidae). In addition, the material provides an opportunity to update and revise existing descriptions of known but poorly described or figured species. We illustrate this with two species – *Harmothoe anderssoni* Bergström, 1916 which is here redescribed and *Aphelochaeta longisetosa* (Hartmann-Schröder, 1965) where the holotype (ZMH- P15068) is imaged for the first time.

The availability of such data and collections to the research community is also an important part of the curatorial function of NHM London, therefore large number specimens (*N* = 18,015) were formally accessed into museum’s collection (http://data.nhm.ac.uk/) following Darwin Core format and the data are made available here. The vouchering and curation of the specimens is of the utmostimportance as specimens are the ultimate evidence as to what species have been found. This also creates an opportunity for more taxa to be formerly described as funding and interest develops in the region.

## Materials and methods

### Falkland Islands

Falkland Islands are located in the south-western Atlantic, ca. 450 km north-east of Tierra del Fuego. The islands lie close to the edge of the Patagonian continental shelf and are considered to be part of the Magellan region (e.g., Hedgpeth 1969). From a geological point of view the Falklands Plateau and surrounding basins are result of the break-up of the Gondwana supercontinent in the Jurassic period. In terms of hydrography, there is a strong influence of Falklands Current which derives from the west wind drift around the Southern Ocean and an anticyclonic ring current that is formed around the Falkland Islands.

### Field sampling protocol

**Figure 1.**
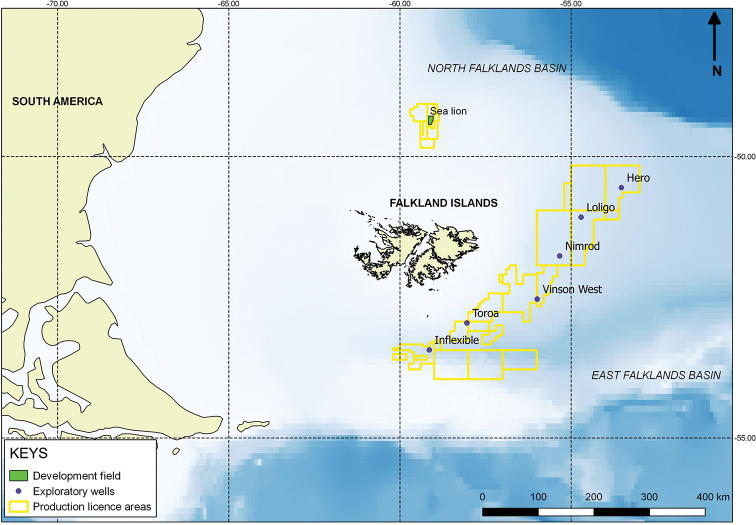
Map showing exploratory oil field (Sea Lion) in North Falklands Basin and exploratory oil wells in East Falklands Basin from which samples in this study were collected.

Samples were collected during contractor-led environmental impact assessment surveys carried out in 2009, 2011, and 2012 (Fig. 1). Two contractors were involved in the field sampling and sampling design (Fugro GB Marine Limited was responsible for 2009 survey and Gardline Limited for 2011 and 2012 survey), below is a summary of methodology used based on available field logs:

1. 2009 survey concentrated on four exploration wells: Toroa (ca. 600 m depth), Nimrod (ca. 1300 m depth), Loligo (ca. 1400 m depth) and Endeavour (ca. 1300 m depth). These wells are located in the East Falkland Basin, southeast to east off the Falkland Islands (see map in Fig. 1). In total 22 stations were successfully sampled, with up to two replicates collected at each station (see, DarwinCore file in Suppl. material 1 for details).
2. 2011 survey concentrated on four exploration wells: Hero (ca. 1800 m depth), Loligo (ca. 1300 m depth), Vinson (ca. 1500 m) and Inflexible (ca. 1800 m depth). These wells are located in East Falkland Basin, southeast to east off Falkland Islands (see map in Fig. 1). In total 18 stations were successfully sampled, with two replicates collected at each station (see Darwin Core file in Suppl. material 1 for details).
3. 2012 survey concentrated on the single exploration oil field – Sea Lion, at depths of ca. 450 m. Sea Lion field is located in North Falkland Basin, north of Falkland Islands (see map in Fig. 1). In total 83 stations were successfully sampled, with three replicates collected at each station (see DarwinCore in Suppl. materials 2, 3 for details).

Most samples were collected using a USNEL box core (area 0.25 m^2^), but in some cases Van Veen Grab sampler was deployed, where the use of boxcorer was prevented (see DarwinCore in Suppl. materials for details). The volume of sampled sediment was not always specified in the field logs and where specified, it varied considerably from 10 to 35 cm of top horizon removed and sieved. Additionally, subcores were taken in some instances for chemical and sedimentary analysis. All samples were sieved on a 0.5 mm mesh sieve. Specimens were fixed in 4% formalin and transferred into 70% ethanol for long-term storage.

### Laboratory identification (carried out at NHM)

Leica MZ6 and DM5000 stereo and compound microscopes were used to examine polychaete specimens. Images of these specimens were captured using a Zeiss V.20 and AxioCam HRc, and a Leica DFC 480 dedicated camera system connected to the DM5000. Photoshop software was used to edit photographs and to compile plates. A camera lucida system was used to draw the specimens. Helicon focus software (HeliconSoft 2018) was used to create composite images of specimens taken at different focal points. The software combines such images to create a clear and focused final image. Specimens of *Harmothoe anderssoni* and *Prosphaerosyllis modinouae* sp. nov. were also examined using a FEI QANTA FEG 650 (FEI, USA) SEM. These specimens were firstdehydrated to 100% alcohol before being critically point dried, mounted on stubs, and coated with gold-vanadium using a sputter coater.

## Results

### Species determinations

From the material examined, in total 233 species were determined following morphological examinations during the contract work, with 191 species included in on-line taxonomic guide (see the checklist). Of these, 123 species were determined from exploration wells in the East Falklands Basin and 110 species from the Sea Lion exploration field in the North Falklands Basin. Voucher specimens, the best-preserved representatives of each taxon, were selected to represent each morphospecies found in the material. Vouchers were not necessarily in “pristine condition”, and not all characters were always observed or determined with certainty. Thus, polychaete morphospecies determined fall into three groups:

1. “The Knowns” – these are species known to science and described from “the region” (Magellanic province, sub-Antarctic islands and parts the Southern Ocean, deep-parts of the Atlantic or Pacific Oceans were also sometimes considered). These species were assigned to known Linnean-named species (e.g., Onuphis pseudoiridescens Averincev, 1972). “The Knowns” represent a small group of the available Falkland Islands polychaete fauna, with 37 species, representing only 16% of our total (available) material.
2. “The Uncertain” – the status of such species is currently uncertain and these are designated “cf.” The reasons of uncertainty were usually as follows: i) specimens were too damaged and/or incomplete, therefore not all characters could be observed; ii) species were considered cosmopolitan having a wide geographic and/or bathymetric distribution, with the type locality outside of “the region” as defined earlier (usually the type locality is in Northern Europe); and/or iii) the original description is of little value (too short, too general, based on incomplete or damaged specimens, unaccompanied by drawings etc.). The type specimens housed at NHM collection were always examined, but those housed by other institutions were not always requested due to time constraints of the initial phase of this project and should be examined in the future work. Only 30 species were assigned to this category, ca. 13% of the diversity.
3. “The Unknowns” (new species) – includes species which were recognised as new to science and ca. 70% of species fall into this category. These taxa are currently assigned to morphospecies name (e.g., Leitoscoloplos sp. 1) and some will be formally described. However, many of these species are represented by only one specimen and/or the specimens are damaged or too incomplete to provide a full diagnosis. It is hoped that future sampling work will provide better examples of such species, enabling their formal description.

An on-line taxonomic guide was created for 191 morphospecies using the Scratchpad platform (Smith et al. 2012) and links to preliminary informal descriptions and images can be found in the check list in this publication. The taxonomic guide itself is an identification tool and its aim is not solving taxonomic problems. These are merely highlighted (where possible), stressing the need for future work by taxonomic experts in particular polychaete groups. Finally, it is important to note, that only morphological examination has been carried out so far as the specimens were not suitable for molecular work. Ultimately, recent taxonomic research has shown that morphological examination has its limits and molecular work is often needed to resolve taxonomic problems (Nygren 2014).

The remainder of this publication concentrates on re-descriptions or formal descriptions of new species, where material was available in condition that is conductive to observation of important taxonomic characters. Whilst this work concentrates on a fraction of the ‘unknowns’, it represents a useful advance, coupled with the significant effort to make raw data and vouchers for the entire collection available for loan. Many known species described in the late 19^th^ and early 20^th^ centuries suffer from poor descriptions and lack of images. Thus, we provide an up to date description, accompanied by photographs and SEM images for *Harmothoe anderssoni* Bergström, 1916 a commonly reported species from the area, with confused taxonomic history to highlight the potential of material presented in this publication. Similarly, Hartmann-Schröder (1965) described *Aphelochaeta longisetosa* but did not provide any figures. We have taken the opportunity to provide images of the holotype as well as specimens of the species collected from the Falkland Islands.

### Accession of the specimens into NHM London collection

Voucher specimens (*N* = 161) from East Falkland Basin surveys were formally accessioned into museum’s collection and the data are here made available via Darwin Core Database (Suppl. material 1). We also undertook accession-ready re-sorting of the material from the North Falkland Basin (Sea Lion oil field). As a result, 15,394 specimens were accessioned at species level (Suppl. material 3) and 2,460 specimens were accessioned at family level (Suppl. material 2) due to time constraints on the re-sorting. All together 18,015 specimens were accessioned to date out of total of ca. 25,000 specimens available.

## Systematic accounts

### Polynoidae Kinberg, 1856

#### Harmothoe anderssoni

Taxon classificationAnimaliaPhyllodocidaPolynoidae

Bergström, 1916

B39AD3AA-3CE6-517D-B4D9-EA0424947A1B

Figures 2 , 3 , 4

Harmothoe anderssoni Bergström, 1916: 286; Monro 1930: 57.
Eunoe anderssoni (Bergström, 1916): Monro 1936: 90; Hartman 1953: 31.
Hermadion anderssoni (Bergström, 1916): Fauvel 1950: 754.
Harmothoe (Eunoe) anderssoni (Bergström, 1916): Hartmann-Schröder and Rosenfeldt 1990: 92; Hartmann-Schröder and Rosenfeldt 1992: 89.

##### Materials.

Sample 40MFC, 442 m, -49.3403947, -59.0845558, coll. 23/04/2012, Ind. 1, NHM.2018.24629. Sample 75MFC, 438 m, -49.3383880, -59.1262342, coll. 22/03/2012, Ind. 1, NHM.2018.21524. Sample 28MFA, 452 m, -49.3044189, -59.0852225, coll. 23/04/2012, Ind. 1, NHM.2018.24377. Sample 30MFB, 456 m, -49.3039703, -59.0302064, coll. 25/04/2012, Ind. 1, NHM.2018.24424. Sample 35MFB, 450 m, -49.3221858, -59.0573711, coll. 15/04/2012, Ind. 1, NHM.2018.24490. Sample 36MFB, 450 m, -49.3219581, -59.0298531, coll. 25/04/2012, Ind. 1, NHM.2018.24508. Sample 38MFB, 434 m, -49.3408178, -59.1396133, coll. 23/04/2012, Ind. 1, NHM.2018.24547. Sample 42MFC, 433 m, -49.3590078, -59.1668389, coll. 14/04/2012, Ind. 1, NHM.2018.24664. Sample 53MFC, 444 m, -49.3751330, -59.1116004, coll. 25/03/2012, Ind. 1, NHM.2018.24908. Sample 67MFB, 442 m, -49.2889470, -59.1102897, coll. 19/03/2012, Ind. 1, NHM.2018.25176. Sample 75MFB, 438 m, -49.3383880, -59.1262342, coll. 22/03/2012, Ind. 1, NHM.2018.21519. Sample 77MFA, 432 m, -49.3511630, -59.1260079, coll. 22/03/2012, Ind. 1, NHM.2018.21547. Sample 19MFA, 443 m, -49.2870544, -59.1680519, coll. 17/04/2012, Ind. 2, NHM.2018.11562–11563. Sample 82MFA, 443 m, -49.2614970, -59.1672448, coll. 16/03/2012, Ind. 2, NHM.2018.12638–12639. Sample 56MFC, 450 m -49.2451170, -59.0544011, coll. 18/03/2012, Ind. 3, NHM.2018.13437–13439.

##### Description.

**Figure 2.**
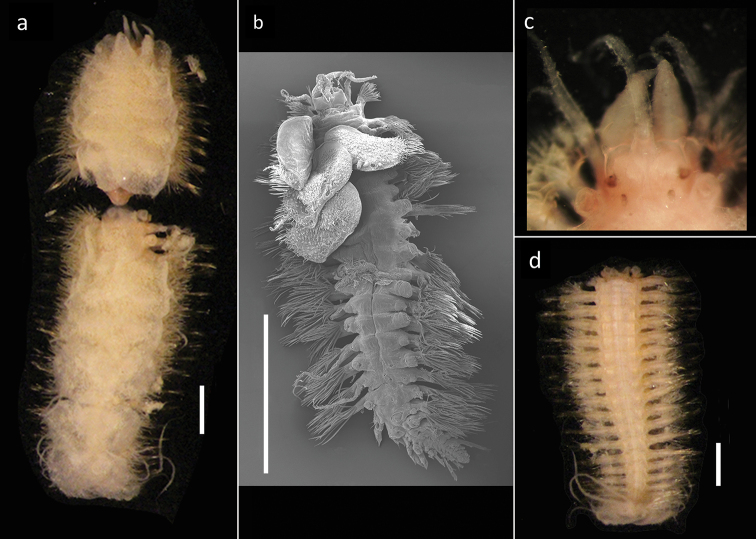
*Harmothoe anderssoni* (voucher NHM.2018.24629, unless stated otherwise) **a** complete specimen (in two fragments) in dorsal view **b** SEM micrograph of the complete specimen in dorsal view (voucher, NHM.2018.21524) **c** detail of prostomium in dorsal view **d** pygidium with pygidial cirri in ventral view. Scale bars: 1 mm (**a, d**); 2 mm (**b**).

Voucher, NHM.2018.24629, a complete specimen (in two fragments) with 35 segments (Fig. 2a), 7.5 mm long and 2 mm wide, including parapodia, but excluding chaetae. Voucher (SEM specimen on stub), NHM.2018.21524, a complete specimen with 35 segments 5 mm long and 1.5 mm wide, including parapodia, but excluding chaetae (Fig. 2b).

Prostomium bilobed, with cephalic peaks (Fig. 2b, c); ceratophore of median antenna in anterior notch, style of median antenna papillate, slightly inflated subdistally, then abruptly tapering; lateral antennae inserted ventrally with styles papillate, tapering. Anterior pair of eyes situated dorsolaterally on widest part of prostomium, posterior pair dorsally near hind margin of prostomium. Palps papillate, tapering.

Tentaculophores inserted laterally to prostomium, each with notochaetae; styles of dorsal and ventral tentacular cirri papillate, slightly inflated subdistally, then abruptly tapering; the dorsal cirri longer than ventral cirri. Second segment with first pair of elytra, biramous parapodia, and long buccal (ventral) cirri.

**Figure 3.**
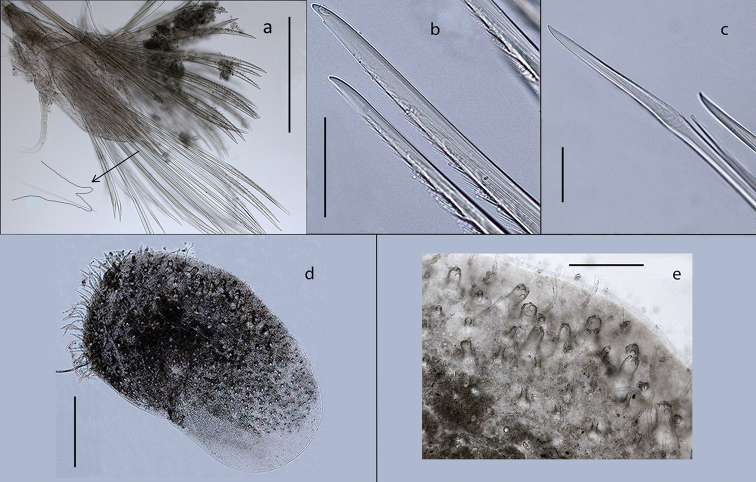
*Harmothoe anderssoni* (voucher, NHM.2018.24629) **a** mid-body parapodium, insert showing line drawing of supra-acicular neuropodial lobe **b** bidentate neurochaetae **c** unidentate neurochaetae **d** mid-body elytron showing elongated marginal papillae **e** detail of elytral surface from mid-body elytron showing multifid macrotubercules. Scale bars: 500 µm (**a, d**); 50 µm (**b, c**); 200 µm (**e**).

**Figure 4.**
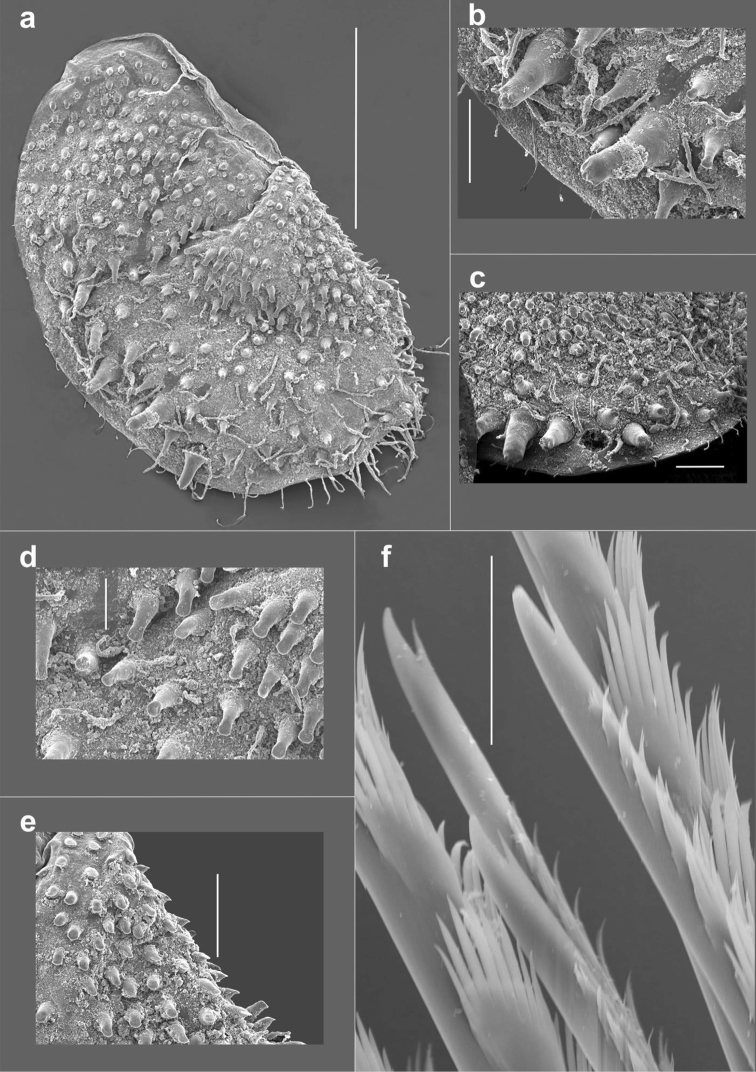
*Harmothoe anderssoni* (SEM micrograph), (voucher, NHM.2018.21524) **a** detached elytron from mid-body **b** detail of the multifid macrotubercles **c** detail of the posterior elytral margin **d** detail of microtubercules from the mid-section of the elytra **e** detail of conical microtubercules near the anterior elytral margin **f** detail of bidentate neurochaetae. Scale bars: 500 µm (**a**); 100 µm (**b, c, e**); 50 µm (**d**); 20 µm (**f**).

Fifteen pairs of elytra covering dorsum, on segments 2, 4, 5, 7, on alternating segments until segment 23, then on segments 26, 29 and 32; all elytra present in voucher specimen (NHM.2018.24629) (Fig. 2a). Outer lateral margin of elytra with dense fringe of long, filiform papillae (Figs 3d; 4a), such papillae also cover elytral surface in between tubercles. Elytral surface covered with 3 forms of tubercles (Figs 3e; 4a–e): small and conical microtubercules confined to anteriormostmargin of elytra (form 1) begin to increase in size, becoming cylindrical to conical, distally somewhat truncated (form 2) and this form covers most of elytral surface; posteriormost margin of elytra with several (8–10) cylindrical to conical distally multifid macrotubercles (form 3).

Parapodia biramous (Fig. 3a); notopodia with elongate acicular lobe; neuropodia larger than notopodia, with short slender supracicular process (Fig. 3a, insert) tips of noto- and neuro-acicula penetrating epidermis. Cirrigerous segments with indistinct dorsal tubercules and cylindrical cirrophores, styles of dorsal cirri very long (slightly longer than neurochaetae), slender, papillate, tapering. Ventral cirri from chaetiger 2 where long and basally inserted; on subsequent chaetigers ventral cirri short, smooth, tapering, medially inserted on neuropodia. Nephridial papillae distinct from chaetiger 6 till end of the body.

Notochaetae stouter than neurochaetae. Notochaetae curved, with distinct rows of spines on convex side and blunt, unidentate tip; numerous, arranged in ca. five rows, increasing in length with each row (Fig. 3a). Neurochaetae uni- and bidentate (Figs 3b, c; 4f); lower and mid neurochaetae unidentate, falcate, subdistally with spines; upper neurochaetae few, bidentate with slender secondary tooth (often abraded), subdistally with spines.

Pygidium with a pair of very long (reaching back over ten segments), thin, papillate anal cirri (Fig. 2d).

##### Remarks.

During preliminary assessment of Falkland Islands specimens these were assigned to *Harmothoe* due to presence of bidentate neurochaetae, as well as other generic characters such short body, 15 segments with elytra, prostomium with cephalic peaks and neuropodia with supra-acicular lobes. However, this species did not match any described or recently reviewed species of *Harmothoe* from the Southern and South Atlantic Oceans (Barnich et al. 2006; Barnich and Fiege 2009; Barnich and Fiege 2010; Barnich et al. 2012; Miranda and Brasil 2014). The form of elytra was, however, consistent with that described for *Eunoe anderssoni*, a Southern Ocean species, which has a confused taxonomic history. It was originally placed in genus *Harmothoe* by Bergström (1916), referred to as *Eunoe* by Monro (1936), moved to *Hermadion* by Fauvel (1950) and again considered *Eunoe* by Hartman (1966). Our understanding is that the reports of *Polynoe hirsuta* from Chile by Ehlers (1901) are misidentified specimens of *H. anderssoni*, given they were recognised as such by Bergström (1916) and Fauvel (1950). Given that Ehlers’ specimens are not available for examination we prefer not to include these in formal synonymy.

The genera *Eunoe and Hermadion* share some characters with *Harmothoe* such as a short body and the presence of 15 elytragerous segments (see e.g., Barnichand Fiege 2009, 2010; Bock et al. 2010), but while prostomial cephalic peaks and neuropodial supracicular lobes are present in *Harmothoe* and *Eunoe*, these are lacking in *Hermadion* (see Barnich and Fiege 2006). However, *Eunoe* supposed to have only unidentate neurochaetae (see e.g., Barnich and Fiege 2009, 2010), while supra-acicular neurochaetae in Falkland Island specimens are clearly bidentate. Unfortunately, Bergström (1916) did not comment on the form of neurochaetae in detail, but subsequently both unidentate and bidentate neurochaetae were reported in *Eunoe anderssoni* by Monro (1936), Hartman (1953) and Hartmann-Schröder and Rosenfeldt (1992). Monro (1936) commented that three out of six specimens had unidentate chaetae only, but in our experience, the slender secondary tooth becomes easily abraded, which may erroneously lead to the conclusion that all neurochaetae are unidentate. The original placement of this species in genus *Harmothoe* is therefore justified and is referred to here as *Harmothoe anderssoni*. It is important to stress that this decision is driven by practical ease of identification rather than phylogenetic position of this species. Recent molecular work on Polynoidae suggests paraphyly of *Harmothoe* (e.g., Norlinder et al. 2012) and the current designation of polynoid genera is likely problematic. Here, we do not attempt to solve systematic difficulties, but provide new information on *Harmothoe anderssoni* with photographs, high quality SEM images (see also Hartmann-Schröder and Rosenfeldt 1992) and detailed description of this species based on material from Falkland Islands.

##### Distribution.

This species is known from Adélie Coast, Kerguelen Islands, South Georgia, South Orkneys and the Antarctic (Hartmann-Schröder and Rosenfeldt 1992). Here, we record this species from the North Falklands Basin, ca. 450 m depth.

### Syllidae Grube, 1850

#### Prosphaerosyllis modinouae

Taxon classificationAnimaliaPhyllodocidaPolynoidae

Neal & Paterson sp. nov.

D4382279-AE65-5258-BC05-E2AAA5D4AD57

http://zoobank.org/4FE5FE0D-7909-40C1-A8AE-F641192A6048

**Figure 5.**
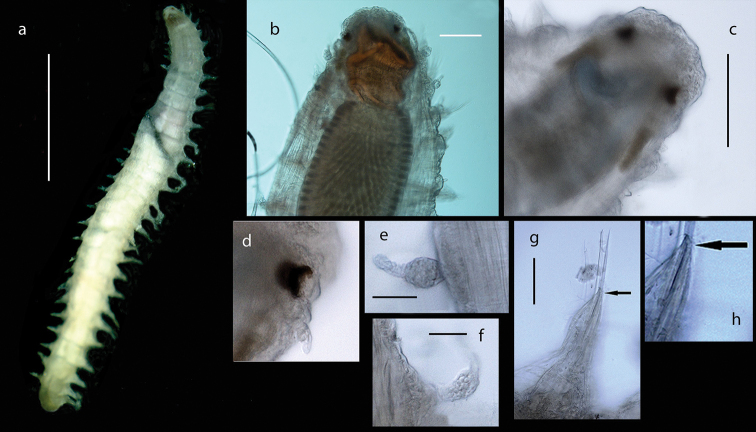
*Prosphaerosyllis modinouae* sp. nov. (paratype, NHM.2018.24386) **a** complete specimen in dorsal view **b** detail of anterior end in dorsal view **c** detail of prostomium and palps in dorsal view **d** detail of eyes **e** anterior dorsal cirrus **f** posterior dorsal cirrus **g** posterior parapodium (arrow marking acicula) **h** detail of acicula. Scale bars: 1000 µm (**a**); 100 µm (**b, c**); 25 µm (**e, f**); 50 µm (**g**).

**Figure 6.**
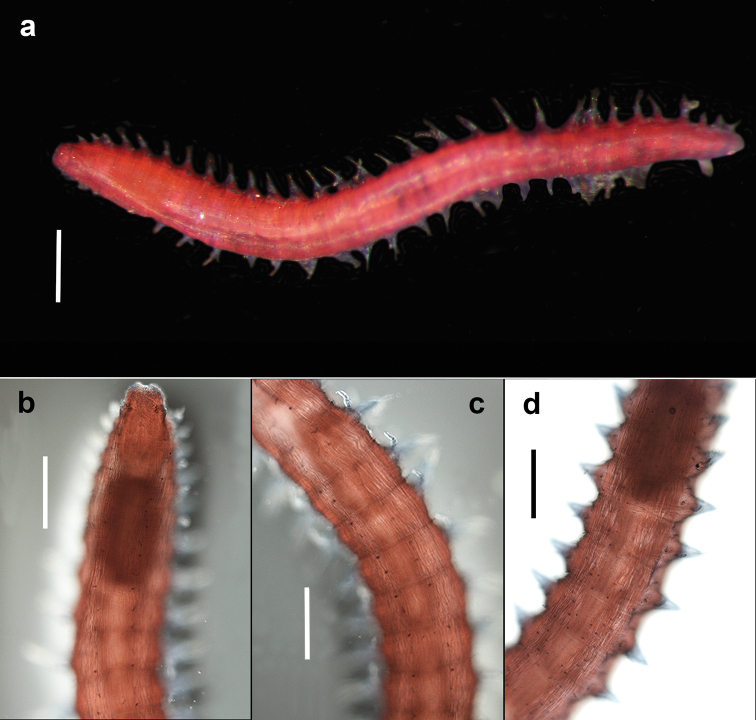
*Prosphaerosyllis modinouae* sp. nov. (holotype, NHM.2018.25100; specimen Shirla-stained) **a** complete specimen in dorsal view **b** pattern of dorsal body papillae (= darkly stained dots) in anterior end **c** pattern of dorsal body papillae (= darkly stained dots) in mid-body segments **d** pattern of ventral papillae (= darkly stained dots). Scale bars: 500 µm (**a**); 200 µm (**b, d**).

**Figure 7.**
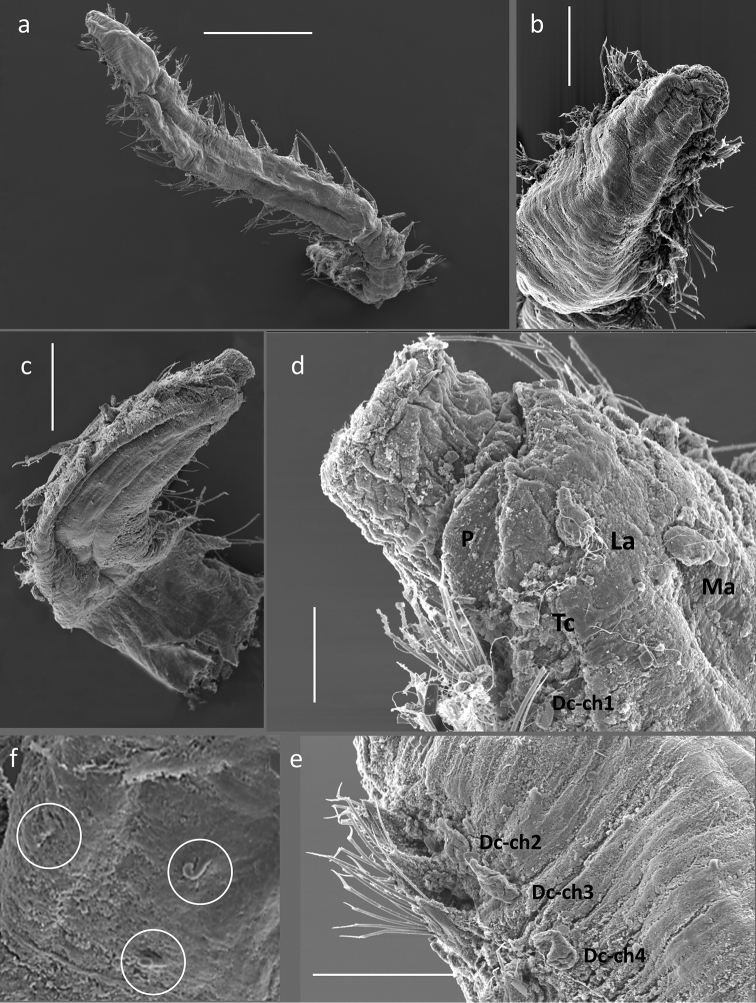
*Prosphaerosyllis modinouae* sp. nov. (SEM micrograph) **a** specimen (paratype, NHM.2018.24386) in dorsal view **b** anterior end in dorsal view **c** paratype (NHM.2018.24236) in dorso-lateral view **d** detail of prostomium of paratype (NHM.2018.24236) with palps (P), median antenna (Ma), lateral antennae (La), tentacular cirrus (Tc) and dorsal cirrus (Dc) **e** detail of dorsal cirri with those on chaetigers 2–4 (paratype, NHM.2018.24386) **f** pattern of dorsal body papillae (marked by white circles) (paratype, NHM.2018.24386). Scale bars: 500 µm (**a**); 100 µm (**b, e**); 200 µm (**c**); 50 µm (**d**).

**Figure 8.**
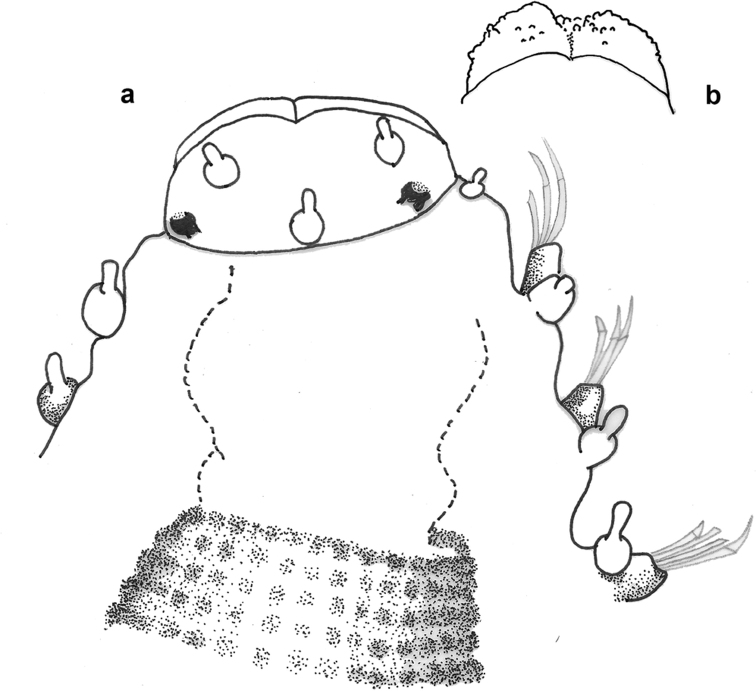
Drawing of anterior end of *Prosphaerosyllis modinouae* sp. nov. in dorsal view (**a**) and detail of palps (**b**).

**Figure 9.**
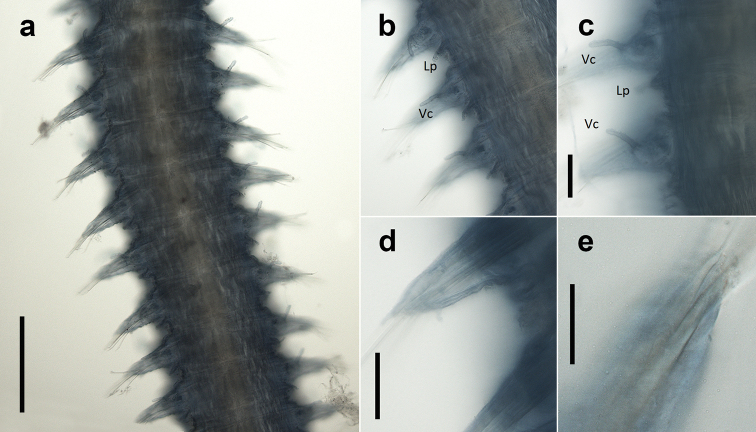
*Prosphaerosyllis modinouae* sp. nov. (holotype, NHM.2018.25100) **a** specimen in partial ventral view **b, c** examples of ventral cirri (Vc) and elongated lateral body papillae (Lp) **d** example of parapodium **e** detail of aciculae. Scale bars: 250 µm (**a**); 50 µm (**c, d**); 20 µm (**e**).

**Figure 10.**
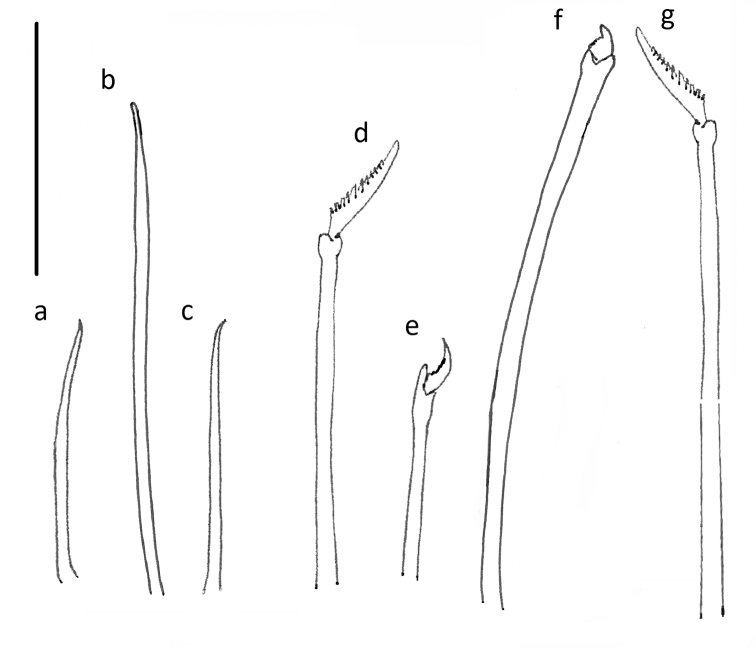
*Prosphaerosyllis modinouae* sp. nov. (paratype NHM.2018.24386) **a** simple dorsal chaeta from anterior chaetiger **b** simple dorsal chaeta from posterior chaetiger **c** simple ventral chaeta from posterior chaetgier **d** dorsalmost falciger from anterior chaetiger **e** ventralmost falciger from anterior chaetiger **f** dorsalmost chaeta from mid body chaetiger **g** falcigers from posterior chaetiger. Scale bar: 50 µm.

**Figure 11.**
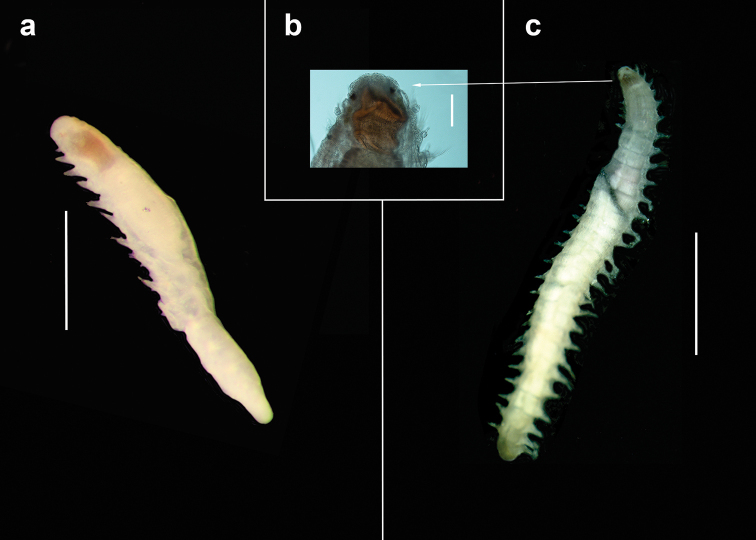
Comparative figure of *Sphaerosyllis palpopapillata* (holotype, ZMH P-20751) (**a**) and *Prosphaerosyllis modinouae* sp. nov. (**b, c**). Scale bars: 1 mm.

Figures 5 , 6 , 7 , 8 , 9 , 10 , 11

##### Materials.

Sample 63MFA, 448 m, -49.2457310, -59.1254934, coll. 17/03/2012, ind. 1, ***holotype*** (NHM.2018.25100). Sample 21MFC, 445 m -49.2866453, -59.1130539, coll. 16/04/2012, ind. 1, ***paratype*** (NHM.2018.24236). Sample 28MFB, 451 m, -49.3044189, -59.0852225, coll. 23/04/2012, ind. 1, ***paratype*** (NHM.2018.24386). **Other materials**: Sample 15MFB, 454 m, -49.2686572, -59.1133764, coll. 16/04/2012, ind. 1, NHM.2018.24080. Sample 25MFA, 447 m, -49.3050428, -59.1677492, coll. 16/04/2012, ind. 1, NHM.2018.24302. Sample 25MFC, 438 m, -49.3050428, Longitude: -59.1677492, coll. 16/04/2012, ind. 1, NHM.2018.24324. Sample 35MFC, 450 m, -49.3221858, -59.0573711, coll. 15/04/2012, ind. 1, NHM.2018.24498. Sample 40MFA, 450 m, -49.3403947, -59.0845558, coll. 23/04/2012, ind. 1, NHM.2018.24605.Sample 41MFA, 439 m, -49.3401736, -59.0570275, coll. 14/04/2012, ind. 1, NHM.2018.24637. Sample 44MFB, 429 m, -49.3585975, -59.1117606, coll. 14/04/2012, ind. 1, NHM.2018.24719. Sample 60MFC, 450 m, -49.2545270, -59.0494527, coll. 19/03/2012, ind. 1, NHM.2018.25062. Sample 64MFC, 447 m, -49.2455380, -59.1060962, coll. 18/03/2012, ind. 1, NHM.2018.25127. Sample 69MFC, 442 m, -49.2887900, -59.1005700, coll. 19/03/2012, ind. 1, NHM.2018.2521. **Comparative materials**: *Prosphaerosyllis kerguelensis*: Kerguelen Islands (off Cumberland Bay), 232 m, -48.750000, -69.233333, holotype, BMNH.85.12.1.155. *Sphaerosyllis palpopapillata*: Antarctic Peninsula, 300 m, -63.27777778, -63.72166667, holotype, ZMH P-20751.

##### Description.

Holotype (NHM.2018.25100) a complete, very small, slender specimen, 4.5 mm long and 0.5 mm wide (at mid-body) for 31 chaetigers. Paratype (NHM.2018.24236), complete specimen 2.8 mm long and 0.4 mm wide (at mid-body) for 31 chaetigers. Paratype (SEM specimen, NHM.2018.24386) complete specimen 3.5 mm long and 0.4 mm wide (at mid-body) for 30 chaetigers. Paratype (SEM specimen, NHM.2018.24236) incomplete specimen 1.2 mm long and 0.25 mm wide for 12 chaetigers. Integument of body appearing smooth under light microscopy (Fig. 5a), upon staining (Fig. 6a–d) and under SEM (Fig. 7a, c, f) sparse papillation detected dorsally with two alternating longitudinal rows of digitiform papillae on each side of the body (= 4 rows in total); palps with some diffused small papillae (Fig. 8b); papillation on body venter diffused (Fig. 6d);parapodial with few tiny papillae (Fig. 9d); slender and elongated lateral body papillae observed in-between parapodia (Fig. 9b, c). Colour in alcohol pale yellow, no pigmentation observed (Fig. 5a).

Prostomium short, wider than long (Figs 5b, c; 8a). Three antennae present (Figs 7d; 8a), all pyriform and very small (difficult to observe under stereo microscope); median antenna positioned near posterior margin of prostomium, ca. 30 µm long; lateral antennae positioned lateromedially on prostomium, smaller than median antenna, ca. 25 µm long (Figs 7d; 8a).

Palps very short, almost entirely obscured by prostomium in dorsal view (Figs 5b, c; 7a–d; 8a) with only their lateral margins visible in dorsal view; fully fused along their length; provided with some small papillae (Fig. 8b). Two pairsof large red eyes present (Figs 5b–d; 8a), in trapezoid arrangement, with anterior and posterior pair close together (almost appearing as a single eye), anterior pair cup-shaped, posterior pair circular; presence of additional eyespots not confirmed.

Proventricle starts between chaetigers 3–4 and ends between chaetigers 6–7, with ca. 20 muscle rows (Figs 5b; 6b). First segment achaetous; with small pair of tentacular cirri, ca. 20 µm long, pyriform (similar to antennae) (Figs 7d; 8a). Pharynx everted in paratype (NHM.2018.24236) (Fig. 7c, d), without terminal papillae, pharyngeal tooth far from anterior margin.

Parapodia uniramous, short but distinct, conical (Fig. 9a–c). Dorsal cirri in all chaetigers, including chaetiger 2 (Figs 7e; 8a) (missing in some parapodia due to damage); dorsal cirri in anterior chaetigers small (but easy to observe, ca. 40 µm in length, Fig. 5e), similar in form to tentacular cirri and antennae, becoming more elongated in posterior chaetigers (ca. 55 µm long, Fig. 5f). Ventral cirri as extremely slender cirriform structures, inserted near the base of neuropodia (Fig. 9a–c), becoming progressively longer posteriorly.

Chaetae often missing (broken off). One simple dorsal chaeta commonly observed (Fig. 10a), present from chaetiger 1; straight and smooth; increasing in size throughout body measuring 30 µm in anterior parapodia, 85 µm in mid-body parapodia and 110 µm in some posterior parapodia (Fig. 10b). Simple ventral chaeta observed only in posteriormost parapodia, slightly sigmoid and smooth (Fig. 10c). Other chaetae compound falcigers (Fig. 10d–g); ca. six per fascicle; all blades unidentate and serrated to greater (Fig. 10d, g) or lesser degree (Fig. 10e, f). Blades of falcigers of varying lengths with greatest length difference between dorsalmost (Fig. 10d) and ventral most (Fig. 10e) chaetae in anterior parapodia, this difference becomes particularly pronounced in mid-body parapodia, where long dorsalmost chaeta (Fig. 10f) bears particularly short blade (as ratio to length of its shaft); length of blades 5–13 µm (total chaetal length 35–60 µm) in anterior chaetigers; 5–13.5 µm (total chaetal length 75–100 µm) in mid-body chaetigers and 12–20 µm (total chaetal length ca.100 µm) in posterior chaetigers (Fig. 10g). Acicula in anterior and posterior chaetigers mostly solitary, acuminate (Figs 5g, h; 9e).

Pygidium broad, rounded, pygidial cirri not observed in any specimens examined (Fig. 5a).

##### Remarks.

Falkland Island specimens were assigned to genus *Prosphaerosyllis* San Martín, 1984 based on morphological characters only as no reproductive specimens were observed. San Martín (2005) emended the generic diagnosis and further distinguished *Prosphaerosyllis* from the similar genus *Sphaerosyllis*, however here we provide a comparison for species in both genera with the type locality inthe southern waters because not all species have been revised. *Sphaerosyllis antarctica*, *S. hirsuta*, *S. sublaevis*, *S. capensis*, *S. dubiosa*, *S. lateropapillata uteae* and *Prosphaerosyllis kerguelensis* (holotype BMNH.85.12.1.155 examined as part of this study) can be easily distinguished from the Falkland Island species due to absence of dorsal cirrus on chaetiger 2. Of species with a dorsal cirrus on chaetiger 2 present (*S. semiveruccosa*, *P. joinvillensis*, *P. capensis chilensis*, *S. brandhorsti* and *P. brachycephala*) the new species can be easily distinguished by having small antennae and short palps (often with only their lateral margins observable in dorsal view, otherwise mostly obscured by prostomium).

Falkland Islands species is most similar to *P. isabellae* De Nogueira, San Martín & Amaral, 2001 described from intertidal depths in Brazil in having short antennae and a sparse distribution of body papillae. However, other than length of the palps, *P. modinouae* sp. nov. can be further differentiated by having all falcigerousblades serrated (these are smooth from mid-body chaetigers in *P. isabellae*), in lacking iridescent inclusions in the dorsal cirri and in the dorsal cirri becoming elongated throughout the body in *P. modinouae* sp. nov. There also appear to be greater differences in length of falcigerous blades in *P. modinouae* sp. nov. (Fig. 10d–g) compared to *P. isabellae* (see DeNogueira et al. 2001; Fukuda et al. 2009). We also suggest that Brazilian specimens from bathyal depths assigned to *P. isabellae* by Fukuda et al. (2009) may in fact represent a different species, even more closely aligned to *P. modinouae* sp. nov. This suggestion is based mainly on much deeper distribution (down to 650 m) reported by Fukuda et al. (2009) compared to 4–7 m depth at the type locality of *P. isabellae*, which was also reported to be associated with coral colonies (De Nogueira et al. 2001). However, specimens of *P. isabellae* were not available for examination as part of this study.

**Figure 12.**
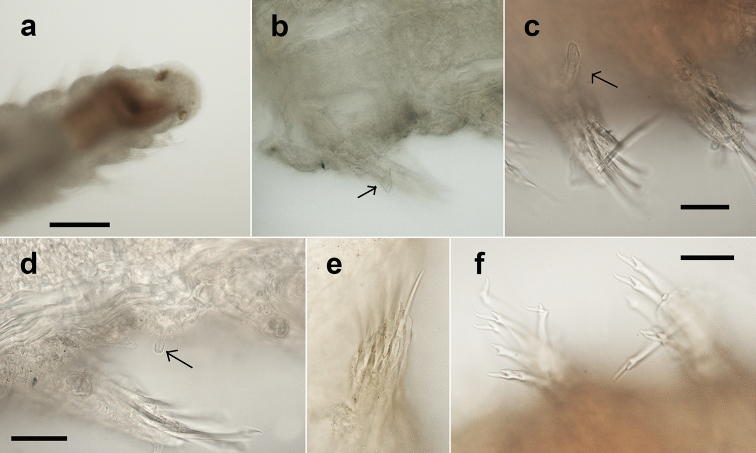
*Sphaerosyllis palpopapillata* (holotype ZMH P-20751) **a** anterior end in dorsal view **b, c** examples of ventral cirri (marked by arrows) **d** example of lateral body papillae (marked by arrow) **e** acicula and simple ventral chaeta **f** falcigers. Scale bars: 1000 µm (**a**); 20 µm (**c, f**), 25 µm (**d**).

Another similar species is *Sphaerosyllis palpopapillata* Hartmann-Schröder & Rosenfeldt, 1992 described from the Antarctic Peninsula, 300 m depth. Unfortunately, the description and drawings provided by Hartmann-Schröder and Rosenfeldt (1992) are of limited value. Therefore, the holotype (ZMH P-20751) was loaned from Zoologisches Museum Hamburg and photographed here for the first time (Figs 11a; 12a–f). The holotype, which is the only known specimen of this species, was found to be a small anterior fragment, with structures such as the antennae now missing andsome chaetae broken off. As a result, *S. palpopapillata* remains a poorly known species, until new material from the type locality becomes available. The holotype (ZMH P-20751) (Fig. 11a) is similar to *P. modinouae* sp. nov. (Fig. 11b, c) in the form of the palps and also possesses the elongated lateral body papillae (Fig. 12d), which were not reported by Hartmann-Schröder and Rosenfeldt (1992), although the rows of dorsal papillae were not confirmed by us. The main differences observed were the form and length of ventral cirri, which are distinctly longer and slender in *S. modinouae* sp. nov. (Figs 9b, c; 12b, c). While both species have unidentate falcigers, their blades in *S. palpopapillata* are shorter (6–7 µm) and similar in size where observed (Fig. 12e, f), but in the new species their lengths are more variable (Fig. 10d–g) as already discussed in comparison with *P. isabellae*. Furthermore, the image provided in the original description (Hartmann-Schröder and Rosenfeldt 1992: fig. 34) suggests that antennae in *S. palpopapillata* are large, not small as in the new species or in *P. isabellae*. Unfortunately, as already mentioned, the antennae have since been lost in the holotype of *S. palpopapillata* and this character cannot be verified.

The lack of detailed descriptions and reliable drawings/images of the known species from the southern waters, complicate the efforts in describing new species. While we believe that observations provided in this study justify the establishment of a new species from the Falkland Islands material, the known species are clearly in need of revision. However, such an undertaking is beyond the scope of this study.

##### Etymology.

This species is dedicated to Yvett Modinou, a passionate science communicator, who inspired the fourth author (BS) to join the NHM London.

##### Distribution.

This species is only known from its type locality, North Falklands Basin, ca. 450 m depth.

### Apistobranchidae Mesnil & Caullery, 1898

#### Apistobranchus jasoni

Taxon classificationAnimaliaPhyllodocidaPolynoidae

Neal & Paterson sp. nov.

C5EC41C2-6CBA-58A1-873B-A1046C8B1AB8

http://zoobank.org/D15234B3-5111-4635-8E05-4F9E6D048361

**Figure 13.**
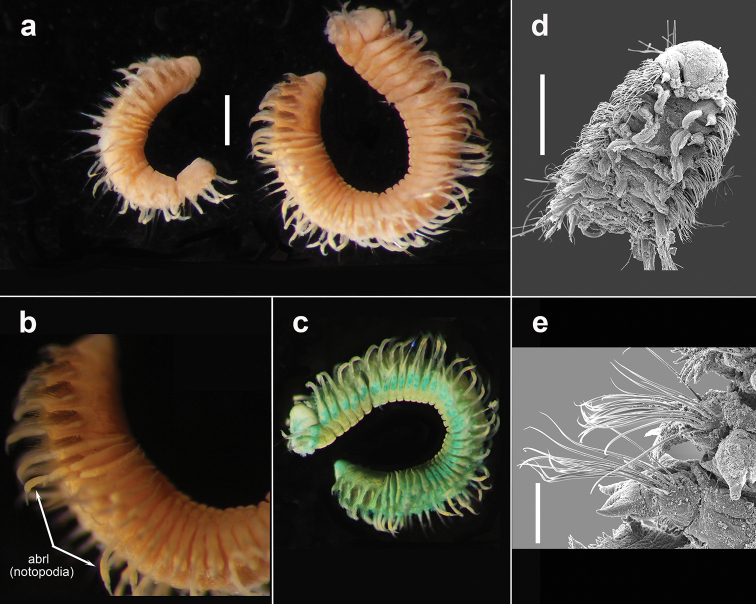
*Apistobranchus jasoni* sp. nov. **a** example of juvenile (paratype, NHM.2018.12713) (left) and adult (holotype, NHM.2018.12712) (right) specimen in lateral view **b** transition between regions with (chaetigers 2–6 and 10 onwards) and without (chaetigers 7–10) acicular branchiae-like lobes (abrl) **c** specimen with Methyl Green stain pattern **d** SEM micrographs of anterior region in dorsal view **e** SEM micrographs of capillary chaetae. Scale bars: 1 mm (**a**); 500 µm (**d**); 100 µm (**e**).

**Figure 14.**
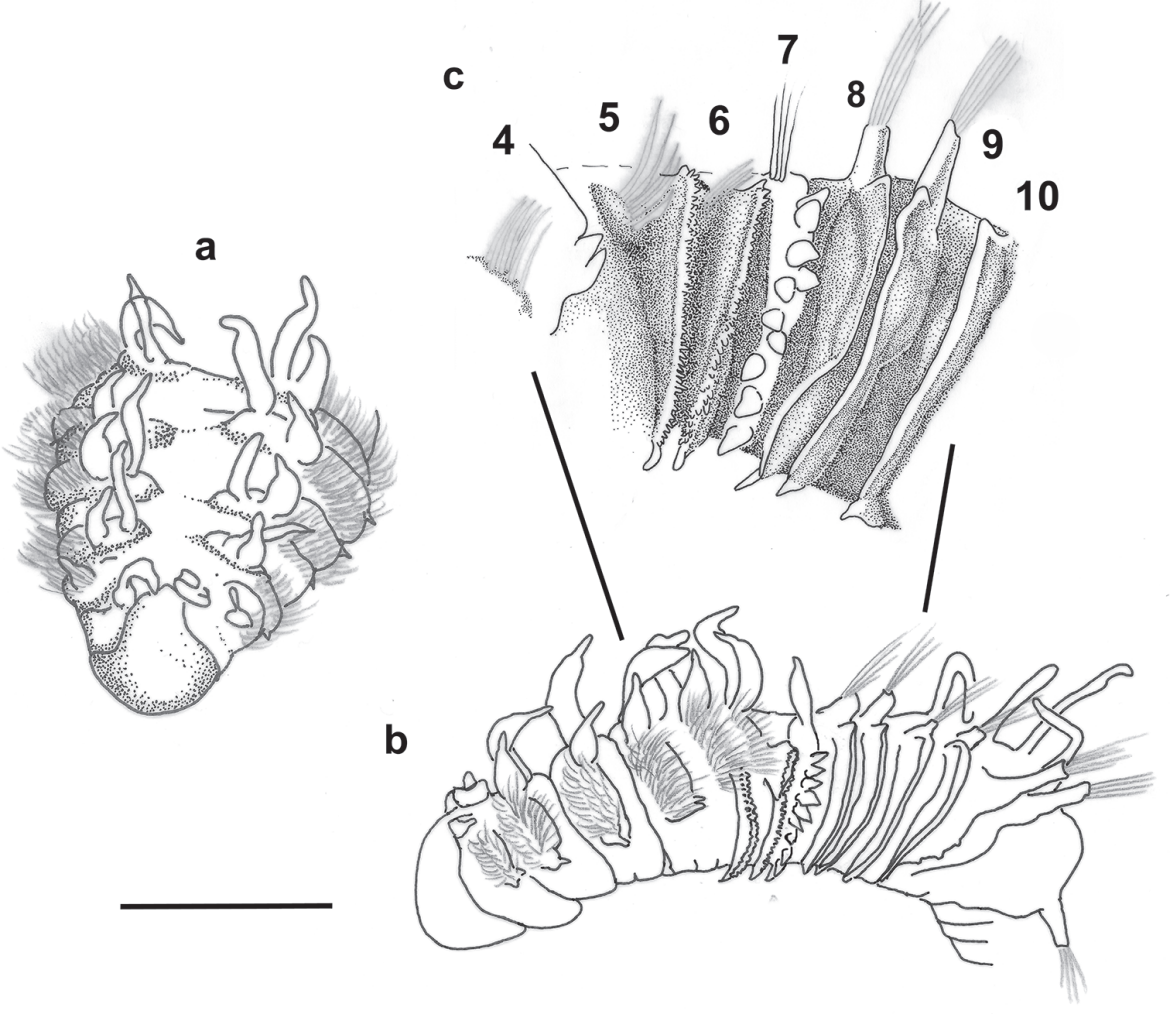
*Apistobranchus jasoni* sp. nov. Illustrations of holotype (NHM.2018.12712) **a** anterior end in dorsal view **b** anterior end in lateral view and **c** detail of chaetigers 4 to 10 (numbered). Scale bar: 1 mm.

**Figure 15.**
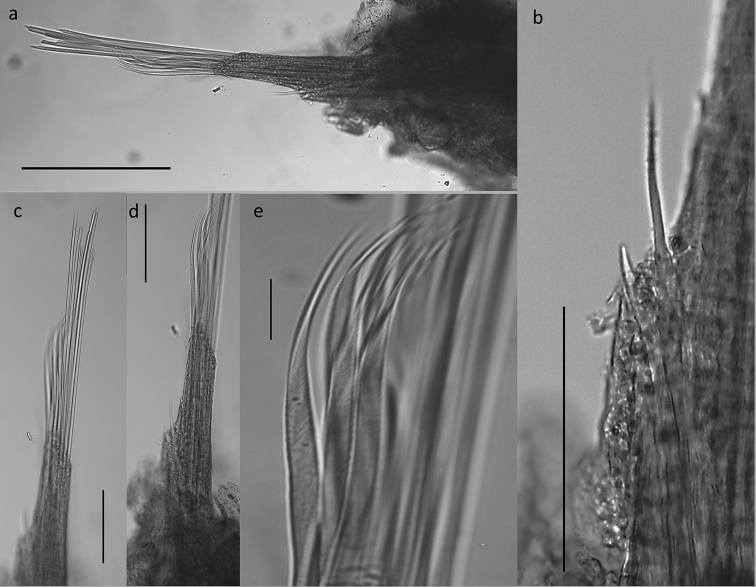
Chaetae of *Apistobranchus jasoni* sp. nov. (holotype NHM.2018.12712) **a** abdominal chaetiger **b** detail of neuropodial acicular chaeta and accompanying capillary **c, d** overview of neuropodial chaetal fascicle with long capillaries with frayed (damaged) tips and short falcate chaetae **e** detail short falcate neurochaetae. Scale bar: 250 µm (**a**); 25 µm (**b**); 100 µm (**c–e**).

Figures 13 , 14 , 15

##### Materials.

Sample 65MFC, 450 m, -49.2584430, -59.1251589, coll. 19/03/2012, ind. 1, ***holotype*** (NHM.2018.12712). Sample 65MFC, 450 m, -49.2584430, -59.1251589, coll. 19/03/2012, ind. 1, ***paratype*** (NHM.2018.12713). **Other materials**: Sample 15MFC, 454 m, -49.2686572, -59.1133764, coll. 16/04/2012, ind. 1, NHM.2018.24086. Sample 19MFB, 448 m, -49.2870544, -59.1680519, coll. 17/04/2012, ind. 1, NHM.2018.24172. Sample 26MFB 449 m, -49.3048414, -59.1402400, coll. 24/04/2012, ind. 1, NHM.2018.24333. Sample 26MFC, 449 m, -49.3048414, -59.1402400, coll. 24/04/2012, ind. 1, NHM.2018.24343. Sample 28MFA, 452 m, -49.3044189, -59.0852225, coll. 23/04/2012, ind. 1, NHM.2018.24371. Sample 35MFA, 450 m, -49.3221858, -59.0573711, coll. 15/04/2012, ind. 1, NHM.2018.24476. Sample 3MFC, 464 m, -49.2326806, -59.1140206, coll. 16/04/2012, ind. 1, NHM.2018.24593. Sample 40MFA, 450 m, -49.3403947, -59.0845558, coll. 23/04/2012, ind. 1, NHM.2018.24601. Sample 43MFA, 430 m, -49.3588058, -59.1392994, coll. 24/12/2012, ind. 1, NHM.2018.24669. Sample 43MFC, 341 m, -49.3588058, -59.1392994, coll. 24/12/2012, ind. 1, NHM.2018.24688. Sample 45MFC, 434 m, -49.3583828, -59.0842219, coll. 23/04/2012, ind. 1, NHM.2018.24740. Sample 59MFB, 448 m, -49.2545600, -59.0688839, coll. 19/03/2012, ind. 1, NHM.2018.25010. Sample 62MFA, 447 m, -49.2487310, -59.1108340, coll. 18/03/2012, ind. 1, NHM.2018.25084. Sample 64MFC, 447 m, -49.2455380, -59.1060962, coll. 18/03/2012, ind. 1, NHM.2018.25121. Sample 66MFA, 445 m, -49.2583220, -59.1058241, coll. 18/03/2012, ind. 1, NHM.2018.25156. Sample 69MFA, 442 m, Latitude -49.2887900, -59.1005700, coll. 19/03/2012, ind. 1, NHM.2018.25198. Sample 70MFB, 446 m, -49.2985060, -59.1150831, coll. 22/03/2012, ind. 1, NHM.2018.25243. Sample 73MFA, 436 m, -49.3416430, -59.1309949, coll. 22/03/2012, ind. 1, NHM.2018.21484. Sample 75MFC, 438 m, -49.3383880, -59.1262342, coll. 22/03/2012, ind. 1, NHM.2018.21523. Sample 79MFA, 446 m, -49.2488410, -59.1867771, coll. 17/03/2012, ind. 1, NHM.2018.21573. Sample 79MFB, 446 m, -49.2488410, -59.1867771, coll. 17/03/2012, ind. 1, NHM.2018.21578. Sample 7MFB, 455 m, -49.2510775, -59.1686567, coll. 17/04/2012, ind. 1, NHM.2018.21597. Sample 80MFC, 445 m, -49.2518660, -59.1722163, coll. 17/03/2012, ind. 1, NHM.2018.21615. Sample 81MFA, 444 m, -49.2618590, -59.1865357, coll. 16/03/2012, ind. 1, NHM.2018.21622. Sample 82MFA, 443 m, -49.2614970, -59.1672448, coll. 16/03/2012, ind. 1, NHM.2018.21638. Sample 8MFB, 459 m, -49.2508764,-59.1411775, coll. 24/04/2012, ind. 1, NHM.2018.21654. Sample 9MFB, 451 m, -49.2506689, -59.1136986, coll. 16/04/2012, ind. 1, NHM.2018.21695. Sample 10MFC, 460 m, -49.2504547, -59.0862200, coll. 25/04/2012, ind. 2, NHM.2018.11380–11381. Sample 12MFA, 453 m, -49.2500069, -59.0312639, coll. 24/04/2012, ind. 2, NHM.2018.11408–11409. Sample 12MFC, 454 m, -49.2500069, -59.0312639, coll. 24/04/2012, ind. 2, NHM.2018.11420–11421. Sample 13MFB, 440 m, -49.2690658, -59.1683544, coll. 17/03/2012, ind. 2, NHM.2018.11426–11427. Sample 15MFA, 455 m, -49.2686572, -59.1133764, coll. 16/04/2012, ind. 2, NHM.2018.11440–11441. Sample 15MFB, 454 m, -49.2686572, -59.1133764, coll. 16/04/2012, ind. 2, NHM.2018.11454–11455. Sample 16MFA, 459 m, -49.2684428, -59.0858878, coll. 25/04/2012, ind. 2, NHM.2018.11472–11473. Sample 24MFC, 453 m, -49.2859825, -59.0305592, coll. 25/04/2012, ind. 2, NHM.2018.11656–11657. Sample 29MFA, 453 m, -49.3041981, -59.0577142, coll. 15/04/2012, ind. 2, NHM.2018.11758–11759. Sample 35MFC, 450 m, -49.3221858, -59.0573711, coll. 15/04/2012, ind. 2, NHM.2018.11822–11823. Sample 3MFB, 464 m, -49.2326806, -59.1140206, coll. 16/04/2012, ind. 2, NHM.2018.11928–11929. Sample 44MFA, 432 m, -49.3585975, -59.1117606, coll. 14/04/2012, ind. 2, NHM.2018.12038–12039. Sample 46MFA, 434 m, -49.3581611, -59.0566836, coll. 14/04/2012, ind. 2, NHM.2018.12066–12067. Sample 47MFC, 444 m, -49.3769958, -59.1665347, coll. 25/03/2012, ind. 2, NHM.2018.12088–12089. Sample 67MFB, 442 m, -49.2889470, -59.1102897, coll. 19/03/2012, ind. 2, NHM.2018.12394–12395. Sample 6MFC, 458 m, -49.2320192, -59.0316156, coll. 25/04/2012, ind. 2, NHM.2018.12440–12441. Sample 71MFC, 449 m, -49.2983550, -59.0956094, coll. 22/03/2012, ind. 2, NHM.2018.12470–12471. Sample 7MFC, 455 m, -49.2510775, -59.1686567, coll. 17/04/2012, ind. 2, NHM.2018.12600–12601. Sample 81MFB, 444 m, -49.2618590, -59.1865357, coll. 16/03/2012, ind. 2, NHM.2018.12622–12623. Sample 90MFB, 440 m, -49.3626660, -59.1204879, coll. 25/03/2012, ind. 2, NHM.2018.12676–12677. Sample 10MFB, 460 m, -49.2504547, -59.0862200, coll. 25/04/2012, ind. 3, NHM.2018.12723–12725. Sample 11MFA, 460 m, -49.2502342, -59.0587417, coll. 15/04/2012, ind. 3, NHM.2018.12741–12743. Sample 16MFB, 459 m, -49.2684428, -59.0858878, coll. 25/04/2012, ind. 3, NHM.2018.12780–12782. Sample 18MFA, 460 m, -49.2679947, -59.0309117, coll. 24/04/2012, ind. 3, NHM.2018.12816–12818. Sample 18MFC, 460 m, -49.2679947, -59.0309117, coll. 24/04/2012, ind. 3, NHM.2018.12822–12824. Sample 25MFA, 447 m, -49.3050428, -59.1677492, coll. 16/04/2012, ind. 3, NHM.2018.12939–12941. Sample 27MFA, 440 m, -49.3046333, -59.1127311, coll. 19/03/2012, ind. 3, NHM.2018.12978–12980. Sample 29MFC, 452 m, -49.3041981, -59.0577142, coll. 15/04/2012, ind. 3, NHM.2018.13035–13037. Sample 49MFA, 448 m, -49.3765856, -59.1114364, coll. 25/03/2012, ind. 3, NHM.2018.13329–13331. Sample 5MFB, 457 m, -49.2322461, -59.0590836, coll. 16/04/2012, ind. 3 NHM.2018.13494–13496. Sample 5MFC, 457 m, -49.2322461, -59.0590836, coll. 16/04/2012, ind. 3, NHM.2018.13503–13505.Sample 11MFB, 455 m, -49.2502342, -59.0587417, coll. 15/04/2012, ind. 4, NHM.2018.13972–13975. Sample 17MFB, 450 m, -49.2679625, -59.0563564, coll. 15/04/2012, ind. 4, NHM.2018.14012–14015. Sample 31MFB, 442 m, -49.3230311, -59.1674461, coll. 15/04/2012, ind. 5, NHM.2018.15034–15038. Sample 6MFB, 458 m, -49.2320192, -59.0316156, coll. 25/04/2012, ind. 5, NHM.2018.15409–15413. Sample 8MFC, 459 m, -49.2508764, -59.1411775, coll. 24/04/2012, ind. 6, NHM.2018.16193–16198.

##### Description.

Holotype (NHM.2018.12712), incomplete specimen, 6 mm long and 1 mm wide for 40 chaetigers (Fig. 13a). Paratype (SEM specimen on stub, NHM.2018.12713), incomplete specimen, 4 mm long and 0.8 mm wide for 22 chaetigers (Fig. 13a). Body strongly curved, broad, flattened across thoracic region, tapering posteriorly; abdominal segments longer, cylindrical. Colour in alcohol dark yellow to pale orange (Fig. 13a, b).

Prostomium bluntly rounded, wider than long, continuing posteriorly as short caruncle terminating in middle of chaetiger 1 (Figs 13d; 14a); palps missing (notobserved); eyes absent; paired nuchal organs prominent, located posterior to the origin of palps (Fig. 13d). Peristomium greatly reduced to lips surrounding mouth.

Notopodia reduced to erect, branchiae-like lobes with internal acicula, present on chaetigers 2–6, then absent on chaetigers 7–10 (Figs 13b; 14b) and then continuing to end of fragment. Chaetigers 1–6 with basally thick, distally tapering interramal cirrus; neuropodia from 7^th^ chaetiger modified, with dense fascicles of pointed capillary neurochaetae. Neuropodia adorned with various lobes and cirri as follow (Fig. 14b): chaetigers 1–3 with single, triangular lobe at ventral-most edge of neuropodium; chaetiger 4 with two lobes on raised ridge; chaetigers 5–6 with thin membranous lamella with numerous (> 30) very small papillae; chaetiger 7 with ten large triangular lobes (5–7 in smaller specimens). Neuropodia of chaetigers 8–11 resembling those of chaetigers 5–6; subsequent (abdominal) neuropodia reduced to long, narrow lobes with few long neurochaetae.

Neurochaetae of first seven chaetigers somewhat stout with simple, gently bent blunt tips (Fig. 13e; arranged in 4–6 palisaded rows (Fig. 13b). Abdominal neurochaetae few (Fig. 15a–e), the main fascicle composed of two types (Fig. 15a): Type 1. Long capillaries, with frayed (damaged?) tips, fewer than ten per fascicle (Fig. 15a, c); Type 2. Short, falcate chaetae, with gently curved, very long thin tips, ca. 6 per fascicle (Fig. 15a, d, e). Additionally, a short, blunt posterior, protruding acicular spine, accompanied by a single short capillary with slender tip, have been observed in abdominal neuropodia (Fig. 15a, b). Pygidium not observed.

##### Methyl Green stain pattern

**(Fig. 13c).** Stain retained on prostomium and peristomium, chaetigers 1–4 do not retain stain (pale stain at the best), chaetigers 5–10 stain lightly interramally, from chaetiger 12 until end of the fragment with stain strongly concentrating on the posterior edge of the neuropodial lamellae, but no patterns of dorsum or ventral surfaces observed.

##### Remarks.

New species belongs to an *Apistobranchus* group in which notopodia are missing on chaetigers 1 and 7–10, with neuropodium on chaetiger 4 multilobed (see Blake 1996). The Falkland Islands species is clearly different from *Apistobranchus glacierae* Hartman, 1978 the only valid species known from the area according to Petti et al. (2007), who clarified the status of *A. glacierae* and *A. gudrunae* Hartmann-Schröder & Rosenfeldt, 1988. In *A. glacierae* the notopodiaare absent on chaetigers 1 and 8 only, unlike *A. jasoni* sp. nov. in which they are absent from chaetigers 1 and 7–10.

It is difficult to distinguish Falkland Islands specimens from *A. ornatus* Hartman, 1965 redescribed by Blake (1996) from the Pacific, which in turn is difficult to distinguish from *A. typicus* (NW Atlantic distribution) other than by methyl green stain (see discussion in Blake (1996)). Falkland Island specimens do not stain uniformly as Blake (1996) reported for *A. ornatus* and no paired ventral line extending from chaetiger 7–8 has been detected. Stain is, however, more pronounced in Falkland Islands specimens then in *A. typicus*, in which a pale reaction is limited to swollen dorsal glandular areas according to Blake (1996). Further, while the posterior protruding spine has been reported in *A. ornatus* (Blake & Petti, 2019), to our knowledge there has been no reports of its accompanying capillary as observed in Falklands specimens (Fig. 15b). Additionally, when comparing specimens with similar number of segments, further subtle differences can be detected and these are summarised in Table 1. Overall, the Falkland Island specimens are smaller and more slender than *A. typicus* and *A. ornatus*, with fewer rows of neurochaetae and fewer lobes on chaetigers 4. We suggest that there is evidence that Falkland Islands’ specimens represent new species.

**Table 1.** Summary of morphological variation in *Apistobranchus jasoni* sp. nov., *A. ornatus* as reported by Blake (1996) and *A. typicus* as reported by Webster and Benedict E (1887).

	Variation in *A. jasoni* sp. nov.	Variation in *A. ornatus*	Distribution reported in original description of *A. typicus*
**Length (mm)**	4–6	4–9	8–12 (probably)
**Width (mm)**	0.8–1	1.4–3	1.5–2
**Number of chaetigers (fragments)**	22–40	22–41	not reported
**No. of palisade rows of neurochaetae**	4–6	4–10 or more	not reported
**No. of neurochaetae in abdomen**	ca. 15	10–15	not reported
**No. of lobes on neuropodium of chaetiger 4**	2–3	2–7	4–6
**No. of lobes on neuropodium of chaetiger 7**	5–10	7–10	10
**branchia-like lobes missing on chaetigers**	7-(9) 10	7(8)-10(11)	at least in 8–10 (not reported)
**Type locality**	SW Atlantic: Falkland Is., ca.450 m	NE Pacific: Western Santa Barbara Channel, shelf/slope depths (up to 1990 m)	NW Atlantic: Maine, 14.5–18 m

##### Etymology.

Species name is dedicated to Jason Huque, husband of the sixth author (CH). Co-incidentally, Jason Islands are also an archipelago in the Falkland Islands.

##### Distribution.

This species is known only from its type locality, North Falklands Basin, at ca. 450 m depth.

### Orbiniidae Hartman, 1942

#### Leitoscoloplos olei

Taxon classificationAnimaliaPhyllodocidaPolynoidae

Neal & Paterson sp. nov.

B01C9DB5-613C-5BAB-AC4E-06B7EEBE8B54

http://zoobank.org/6F3704FD-E05C-44C9-BCC7-A10C82CB60D1

**Figure 16.**
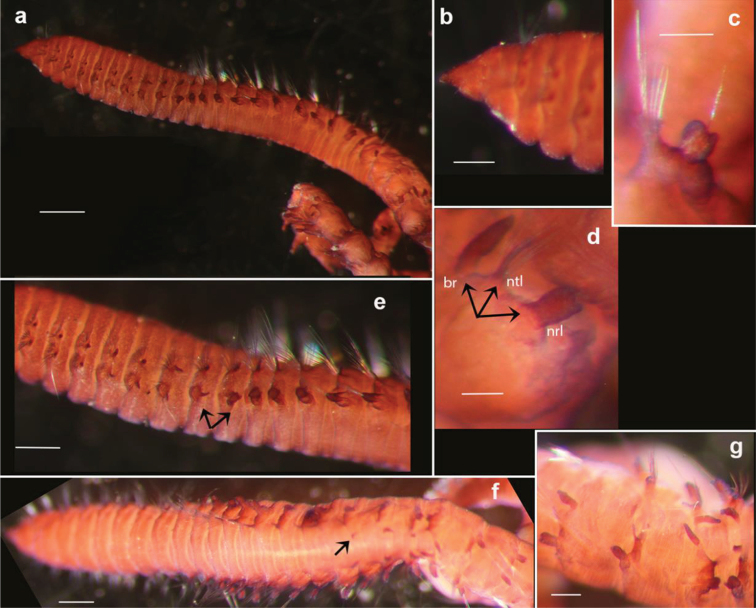
*Leitoscoloplos olei* sp. nov., paratype (NHM.2018.21716) Shirla-stained **a** specimen in lateral view **b** detail of prostomium **c** detail of abdominal chaetiger showing subpodal flange **d** detail of abdominal chaetiger showing branchia (br), notopodial (ntl) and bilobed neuropodial lobes (nrl) **e** detail of transition between thoracic and abdominal segments in lateral view (the last thoracic and first abdominal segment highlighted with arrows) **f** detail of transition to branchiate region in dorsal view (first occurrence of branchiae highlighted with arrow) **g** detail of posterior abdominal segments. Scale bars: 500 µm (**a**); 200 µm (**b, c**); 80 µm (**d**); 250 µm (**e**); 300 µm (**f**); 150 µm (**g**).

**Figure 17.**
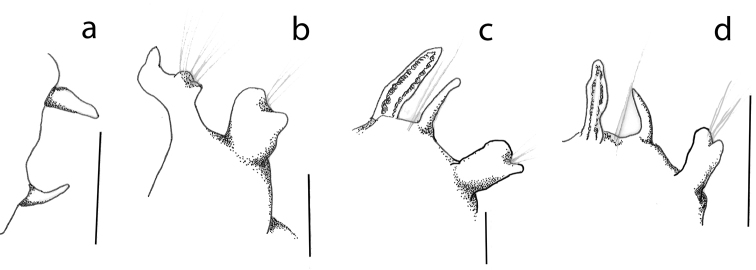
Parapodial sequence of *Leitoscoloplos olei* sp. nov., paratype (NHM.2018.21716) **a** thoracic chaetiger (chaetiger 6) **b** pre-branchiate abdominal chaetiger **c** branchiate abdominal chaetiger **d** posterior branchiate abdominal chaetiger. Scale bars: 1 mm.

**Figure 18.**
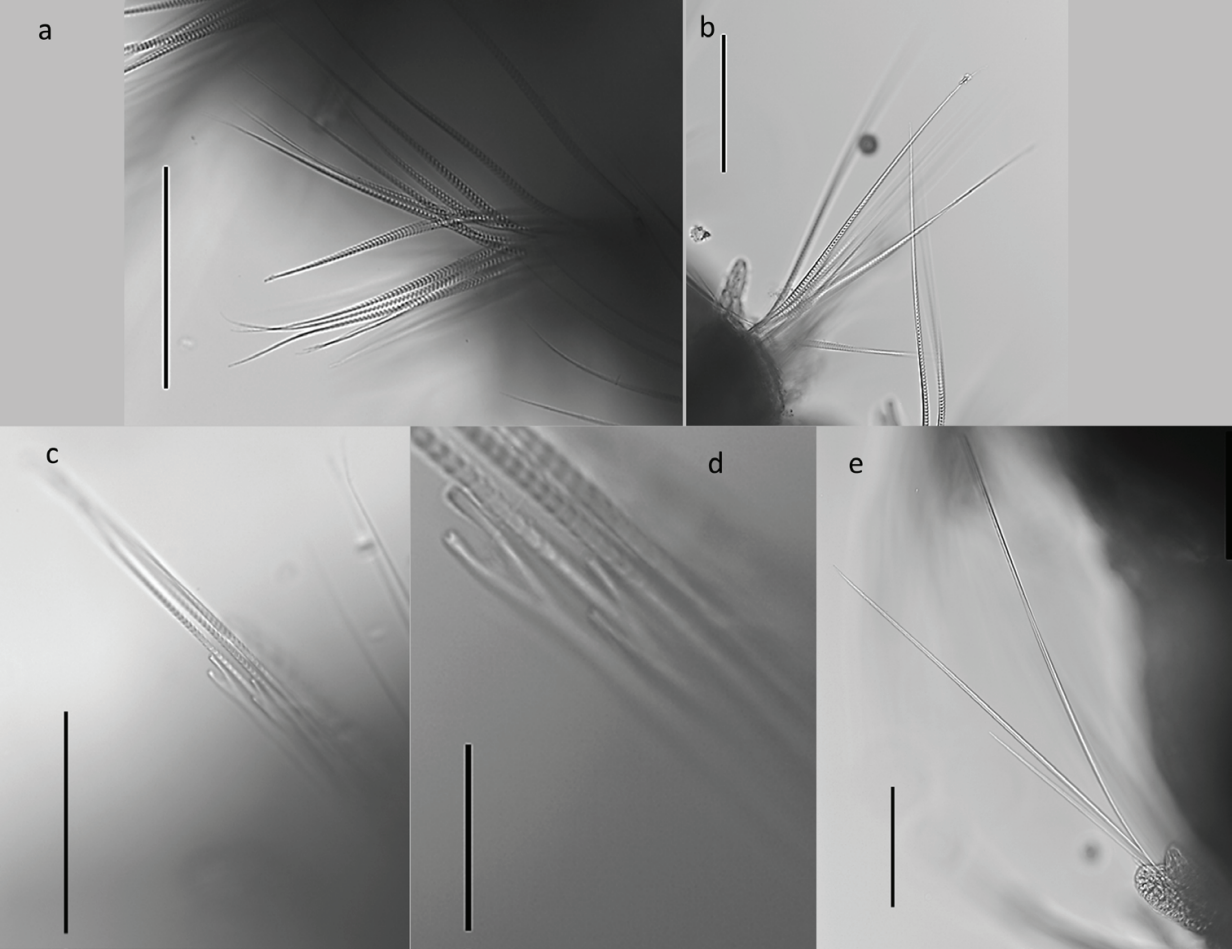
*Leitoscoloplos olei* sp. nov., holotype (NHM.2018.21756) **a** thoracic camerated notochaetae **b** thoracic camerated neurochaetae **c** bundle of neurochaetae, including the camerated capillaries and furcate chaetae **d** detail of furcate chaetae **e** abdominal camerated capillaries. Scale bars: 100 µm (**a–c, e**); 25 µm (**d**).

**Figure 19.**
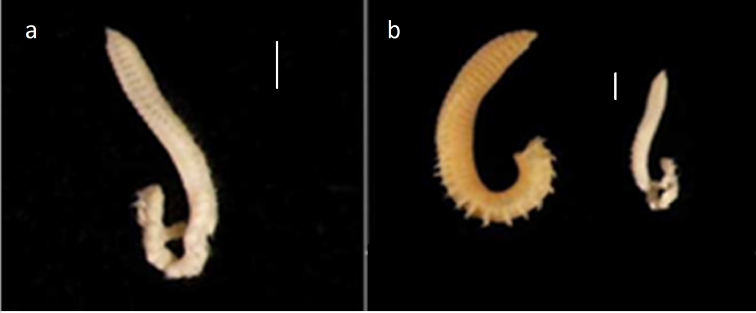
Comparison of *Leitoscoloplos olei* sp. nov. and *L. kerguelensis***a***L. olei* sp. nov., holotype (NHM.2018.21756) in lateral view **b** comparison of *L. olei* sp. nov. and one of the syntypes (BMNH.ZK.1885.12.1.252) of *L. kerguelensis*, both specimens in lateral view. Scale bars: 1mm.

Figures 16 , 17 , 18 , 19

##### Materials.

***Holotype***: Sample 66MFC, 450 m, -49.2584430, -59.1251589, coll. 19/03/2012, ind. 1, NHM.2018.21756. ***Paratypes***: Sample 1MFC, 456 m,-49.2330889, -59.1689586, coll. 18/04/2012, ind. 1, NHM.2018.21716. Sample 60MFA, 450 m, -49.2545270, -59.0494527, coll. 18/03/2012, ind. 4, NHM.2018.14528–14531. **Other materials**: Sample 12MFA, 453 m, -49.2500069, -59.0312639, coll. 24/04/2012, ind. 1, NHM.2018.24036. Sample 13MFC, 440 m, -49.2690658, -59.1683544, coll. 17/03/2012, ind. 1, NHM.2018.24061. Sample 15MFC, 454 m, -49.2686572, -59.1133764, coll. 16/04/2012, ind. 1, NHM.2018.24089. Sample 16MFA, 459 m, -49.2684428, -59.0858878, coll. 25/04/2012, ind. 1, NHM.2018.24100. Sample 19MFA, 443 m, -49.2870544, -59.1680519, coll. 17/04/2012, ind. 1, NHM.2018.24169. Sample 1MFB, 456 m, -49.2330889, -59.1689586, coll. 18/04/2012ind. 1, NHM.2018.24196. Sample 20MFA, 444 m, -49.2868531, -59.1405528, coll. 18/04/2012, ind. 1, NHM.2018.24204. Sample 20MFB, 451 m, -49.2868531, -59.1405528, coll. 23/04/2012, ind. 1, NHM.2018.24211. Sample 22MFB,454 m, -49.2864308, -59.0855553, coll. 23/04/2012, ind. 1, NHM.2018.24249. Sample 23MFA, 450 m, -49.2862100, -59.0580569, coll. 15/04/2012, ind. 1, NHM.2018.24266. Sample 24MFC, 453 m, -49.2859825, -59.0305592, coll. 25/04/2012, ind. 1, NHM.2018.24294. Sample 26MFC, 449 m, -49.3048414, -59.1402400, coll. 24/04/2012, ind. 1, NHM.2018.24347. Sample 27MFA, 440 m, -49.3046333, -59.1127311, coll. 19/03/2012, ind. 1, NHM.2018.24356. Sample 29MFA, 453 m, -49.3041981, -59.0577142, coll. 15/04/2012, ind. 1, NHM.2018.24397. Sample 29MFB, 452 m, -49.3041981, -59.0577142, coll. 15/04/2012, ind. 1, NHM.2018.24403. Sample 36MFC, 450 m, -49.3219581, -59.0298531, coll. 25/04/2012, ind. 1, NHM.2018.24517. Sample 37MFA, 436 m, -49.3410194, -59.1671425, coll. 15/04/2012, ind. 1, NHM.2018.24525. Sample 38MFB, 434 m, -49.3408178, -59.1396133, coll. 23/04/2012, ind. 1, NHM.2018.24549. Sample 39MFB, 436 m, -49.3406094, -59.1120844, coll. 15/04/2012, ind. 1, NHM.2018.24573. Sample 41MFA, 439 m, -49.3401736, -59.0570275, coll. 14/04/2012, ind. 1, NHM.2018.24636. Sample 41MFC, 437 m, -49.3401736, -59.0570275, coll. 14/04/2012, ind. 1, NHM.2018.24644. Sample 43MFA, 430 m, -49.3588058, -59.1392994, coll. 24/12/2012, ind. 1, NHM.2018.24676. Sample 45MFC, 434 m, -49.3583828, -59.0842219, coll. 23/04/2012, ind. 1, NHM.2018.24744. Sample 46MFB, 434 m, -49.3581611, -59.0566836, coll. 14/04/2012ind. 1, NHM.2018.24757. Sample 47MFC, 444 m, -49.3769958, -59.1665347, coll. 25/03/2012, ind. 1, NHM.2018.24780. Sample 48MFA, 425 m, -49.3767939, -59.1389856, coll. 24/04/2012, ind. 1, NHM.2018.24786. Sample 50MFA, 433 m, -49.3763706, -59.0838878, coll. 25/04/2012, ind. 1, NHM.2018.24831. Sample 50MFB, 436 m, -49.3763706, -59.0838878, coll. 25/04/2012, ind. 1, NHM.2018.24837. Sample 50MFC, 436 m, -49.3763706, -59.0838878, coll. 25/04/2012, ind. 1, NHM.2018.24844. Sample 52MFA, 426 m, -49.3947819, -59.1386711, coll. 24/04/2012, ind. 1, NHM.2018.24883. Sample 53MFB, 444 m, -49.3751330, -59.1116004, coll. 25/03/2012, ind. 1, NHM.2018.24903. Sample 53MFC, 444 m, -49.3751330, -59.1116004, coll. 25/03/2012, ind. 1, NHM.2018.24910. Sample 5MFB, 457 m, -49.2322461, -59.0590836, coll. 16/04/2012, ind. 1, NHM.2018.25034. Sample 64MFB, 447 m, -49.2455380, -59.1060962, coll. 18/03/2012, ind. 1, NHM.2018.25119. Sample 64MFC, 447 m, -49.2455380, -59.1060962, coll. 18/03/2012, ind. 1, NHM.2018.25126. Sample 69MFA, 442 m, -49.2887900, -59.1005700, coll. 19/03/2012, ind. 1, NHM.2018.25202. Sample 69MFB, 442 m, -49.2887900, -59.1005700, coll. 19/03/2012, ind. 1, NHM.2018.25207. Sample 72MFC, 432 m, -49.3416800, -59.1407685, coll. 22/03/2012, ind. 1, NHM.2018.21480. Sample 73MFA, 436 m, -49.3416430, -59.1309949, coll. 22/03/2012, ind. 1, NHM.2018.21488. Sample 75MFB, 438 m, -49.3383880, -59.1262342, coll. 22/03/2012, ind. 1, NHM.2018.21521. Sample 75MFC, 438 m, -49.3383880, -59.1262342, coll. 22/03/2012, ind. 1, NHM.2018.21525. Sample 76MFA, 434 m, -49.3513090, -59.1454613, coll. 22/03/2012, ind. 1, NHM.2018.21531. Sample 78MFB, 444 m, -49.2522230, -59.1818433, coll.17/03/2012, ind. 1, NHM.2018.21566. Sample 78MFC, 444 m, -49.2522230, Longitude -59.1818433, coll. 17/03/2012, ind. 1, NHM.2018.21571. Sample 79MFB, 446 m, -49.2488410, Longitude -59.1867771, coll. 17/03/2012, ind. 1, NHM.2018.21584. Sample 79MFC, 446 m, -49.2488410, -59.1867771, coll. 17/03/2012, ind. 1, NHM.2018.21592. Sample 80MFB, 445 m, -49.2518660, -59.1722163, coll. 17/03/2012, ind. 1, NHM.2018.21610. Sample 80MFC, 445 m, -49.2518660, -59.1722163, coll. 17/03/2012, ind. 1, NHM.2018.21619. Sample 81MFB, 444 m, -49.2618590, -59.1865357, coll. 16/03/2012, ind. 1, NHM.2018.21631. Sample 81MFC, 444 m, -49.2618590, -59.1865357, coll. 16/03/2012, ind. 1, NHM.2018.21635. Sample 82MFC, 443 m, -49.2614970, -59.1672448, coll. 16/03/2012, ind. 1, NHM.2018.21649. Sample 9MFC, 451 m, -49.2506689, -59.1136986, coll. 16/04/2012, ind. 1, NHM.2018.21701. Sample 10MFC, 460 m, -49.2504547, -59.0862200, coll. 25/04/2012, ind. 2, NHM.2018.11382–11383. Sample 11MFB, 455 m, -49.2502342, -59.0587417, coll. 15/04/2012, ind. 2, NHM.2018.11400–11401. Sample 13MFB, 440 m, -49.2690658, -59.1683544, coll. 17/03/2012, ind. 2, NHM.2018.11436–11437. Sample 15MFA, 455 m, -49.2686572, -59.1133764, coll. 16/04/2012, ind. 2, NHM.2018.11446–11447. Sample 16MFB, 459 m, -49.2684428, -59.0858878, coll. 25/04/2012, ind. 2, NHM.2018.11494–11495. Sample 18MFB, 460 m, -49.2679947, -59.0309117, coll. 24/04/2012, ind. 2, NHM.2018.11542–11543. Sample 21MFC, 445 m, -49.2866453, -59.1130539, coll. 16/04/2012, ind. 2, NHM.2018.11604–11605. Sample 23MFC, 450 m, -49.2862100, -59.0580569, coll. 15/04/2012, ind. 2, NHM.2018.11646–11647. Sample 24MFB, 453 m, -49.2859825, -59.0305592, coll. 25/04/2012, ind. 2, NHM.2018.11654–11655. Sample 28MFA, 452 m, -49.3044189, -59.0852225, coll. 23/04/2012, ind. 2, NHM.2018.11740–11741. Sample 31MFB, 442 m, -49.3230311, -59.1674461, coll. 15/04/2012, ind. 2, NHM.2018.11782–11783. Sample 32MFC, 438 m, -49.3228297, -59.1399267, coll. 24/04/2012, ind. 2, NHM.2018.11800–11801. Sample 35MFB, 450 m, -49.3221858, -59.0573711, coll. 15/04/2012, ind. 2, NHM.2018.11814–11815. Sample 37MFC, 436 m, -49.3410194, Longitude -59.1671425, coll. 15/04/2012, ind. 2, NHM.2018.11862–11863. Sample 38MFC, 434 m, -49.3408178, -59.1396133, coll. 23/04/2012, ind. 2, NHM.2018.11900–11901. Sample 3MFC, 464 m, -49.2326806, -59.1140206, coll. 16/04/2012, ind. 2, NHM.2018.11936–11937. Sample 40MFA, 450 m, -49.3403947, -59.0845558, coll. 23/04/2012, ind. 2, NHM.2018.11946–11947. Sample 42MFA, 434 m, -49.3590078, -59.1668389, coll. 14/04/2012, ind. 2, NHM.2018.11988–11989. Sample 42MFB, 433, m, -49.3590078, -59.1668389, coll. 14/04/2012, ind. 2, NHM.2018.11996–11997. Sample 43MFB, 341 m, -49.3588058, -59.1392994, coll. 24/12/2012, ind. 2, NHM.2018.12020–12021. Sample 45MFA, 434 m, -49.3583828, -59.0842219, coll. 23/04/2012, ind. 2, NHM.2018.12056–12057. Sample 49MFB, 448 m, -49.3765856, -59.1114364, coll. 25/03/2012, ind. 2, NHM.2018.12118–12119. Sample 49MFC, 448 m, -49.3765856, -59.1114364, coll. 25/03/2012, ind. 2, NHM.2018.12126–12127.Sample 51MFB, 442 m, -49.3949839, -59.1662306, coll. 25/03/2012, ind. 2, NHM.2018.12156–12157. Sample 52MFC, 423 m, -49.3947819, -59.1386711, coll. 24/04/2012, ind. 2, NHM.2018.12184–12185. Sample 54MFA, 426 m, -49.3943581, -59.0835533, coll. 25/04/2012, ind. 2, NHM.2018.12210–12211. Sample 57MFA, 450 m, -49.2419730, -59.0690656, coll. 18/03/2012, ind. 2, NHM.2018.12240–12241. Sample 57MFC, 450 m, -49.2419730, -59.0690656, coll. 18/03/2012, ind. 2, NHM.2018.12242–12243. Sample 59MFA, 448 m, -49.2545600, -59.0688839, coll. 19/03/2012, ind. 2, NHM.2018.12260–12261. Sample 61MFB, 447 m, -49.2487430, -59.1206859, coll. 18/03/2012, ind. 2, NHM.2018.12308–12309. Sample 63MFA, 448 m, -49.2457310, -59.1254934, coll. 17/03/2012, ind. 2, NHM.2018.12326–12327. Sample 63MFC, 448 m, -49.2457310, -59.1254934, coll. 17/03/2012, ind. 2, NHM.2018.12336–12337. Sample 64MFA, 447 m, -49.2455380, -59.1060962, coll. 17/03/2012, 23:30, ind. 2, NHM.2018.12344–12345. Sample 68MFC, 440 m -49.2856340, -59.0958696, coll. 19/03/2012, 07:48, ind. 2, NHM.2018.12410–12411. Sample 6MFB, 458 m, -49.2320192, -59.0316156, coll. 25/04/2012, ind. 2, NHM.2018.12438–12439. Sample 6MFC, 458 m, -49.2320192, -59.0316156, coll. 25/04/2012, ind. 2, NHM.2018.12442–12443. Sample 72MFA, 432 m, -49.3416800, -59.1407685, coll. 22/03/2012, ind. 2, NHM.2018.12478–12479. Sample 77MFA, 432 m, -49.3511630, -59.1260079, coll. 22/03/2012, ind. 2, NHM.2018.12538–12539. Sample 78MFA, 444 m, -49.2522230, -59.1818433, coll. 17/03/2012, ind. 2, NHM.2018.12554–12555. Sample 7MFB, 455 m, -49.2510775, -59.1686567, coll. 17/04/2012, ind. 2, NHM.2018.12590–12591. Sample 82MFA, 443 m, -49.2614970, -59.1672448, coll. 16/03/2012, ind. 2, NHM.2018.12642–12643. Sample 10MFA, 460 m, -49.2504547, -59.0862200, coll. 25/04/2012, ind. 3, NHM.2018.12720–12722. Sample 10MFB, 460 m, -49.2504547, -59.0862200, coll. 25/04/2012, ind. 3, NHM.2018.12726–12728. Sample 15MFB, 454 m, -49.2686572, -59.1133764, coll. 16/04/2012, ind. 3, NHM.2018.12759–12761. Sample 17MFC, 450 m, -49.2679625, -59.0563564, coll. 15/04/2012, ind. 3, NHM.2018.12807–12809. Sample 19MFC, 448 m, -49.2870544, -59.1680519, coll. 17/04/2012, ind. 3, NHM.2018.12849–12851. Sample 27MFC, 440 m, -49.3046333, -59.1127311, coll. 19/03/2012, ind. 3, NHM.2018.12993–12995. Sample 30MFA, 456 m, -49.3039703, -59.0302064, coll. 25/04/2012, ind. 3, NHM.2018.13050–13052. Sample 32MFB, 438 m, -49.3228297, -59.1399267, coll. 24/04/2012, ind. 3, NHM.2018.13074–13076. Sample 35MFC, 450 m, -49.3221858, -59.0573711, coll. 15/04/2012, ind. 3, NHM.2018.13092–13094. Sample 39MFC, 436 m, -49.3406094, -59.1120844, coll. 15/04/2012, ind. 3, NHM.2018.13161–13163. Sample 43MFC, 341 m, -49.3588058, -59.1392994, coll. 24/12/2012, ind. 3, NHM.2018.13257–13259. Sample 44MFB, 429 m, -49.3585975, -59.1117606, coll. 14/04/2012, ind. 3, NHM.2018.13269–13271. Sample 44MFC, 429 m, -49.3585975, -59.1117606, coll. 14/04/2012, ind. 3, NHM.2018.13287–13289. Sample 48MFC, 460 m, -49.3767939, -59.1389856, coll. 24/04/2012, ind. 3, NHM.2018.13326–13328.Sample 4MFB, 457 m, -49.2324667, -59.0865519, coll. 25/04/2012, ind. 3, NHM.2018.13338–13340. Sample 55MFA, 449 m, -49.2452600, -59.0641536, coll. 18/03/2012, ind. 3, NHM.2018.13398–13400. Sample 57MFB, 450 m, -49.2419730, -59.0690656, coll. 18/03/2012, ind. 3, NHM.2018.13455–13457. Sample 58MFC, 453 m, -49.2417960, -59.0496559, coll. 18/03/2012, ind. 3, NHM.2018.13470–13472. Sample 60MFC, 450 m, -49.2545270, -59.0494527, coll. 19/03/2012, ind. 3, NHM.2018.13539–13541. Sample 67MFB, 442 m, -49.2889470, -59.1102897, coll. 19/03/2012, ind. 3, NHM.2018.13620–13622. Sample 70MFA, 448 m, -49.2985060, -59.1150831, coll. 22/03/2012, ind. 3, NHM.2018.13665–13667. Sample 71MFC, 449 m, -49.2983550, -59.0956094, coll. 22/03/2012, ind. 3, NHM.2018.13707–13709. Sample 76MFB, 434 m, -49.3513090, -59.1454613, coll. 23/03/2012, ind. 3, NHM.2018.13773–13775. Sample 76MFC, 434 m, -49.3513090, -59.1454613, coll. 23/03/2012, ind. 3, NHM.2018.13776–13778. Sample 77MFB, 432 m, -49.3511630, -59.1260079, coll. 22/03/2012, ind. 3, NHM.2018.13791–13793. Sample 70MFA, 448 m, -49.2985060, -59.1150831, coll. 22/03/2012, ind. 3, NHM.2018.13665–13667. Sample 71MFC, 449 m, -49.2983550, -59.0956094, coll. 22/03/2012, ind. 3, NHM.2018.13707–13709. Sample 76MFB, 434 m, -49.3513090, -59.1454613, coll. 23/03/2012, ind. 3, NHM.2018.13773–13775. Sample 76MFC, 434 m, -49.3513090, -59.1454613, coll. 23/03/2012, ind. 3, NHM.2018.13776–13778. Sample 77MFB, 432 m, -49.3511630, -59.1260079, coll. 22/03/2012, ind. 3, NHM.2018.13791–13793. Sample 81MFA, 444 m, -49.2618590, -59.1865357, coll. 16/03/2012, ind. 3, NHM.2018.13866–13868. Sample 8MFC, 459 m, -49.2508764, -59.1411775, coll. 24/04/2012, ind. 3, NHM.2018.13902–13904. Sample 90MFC, 440 m, -49.3626660, -59.1204879, coll. 25/03/2012, ind. 3, NHM.2018.13917–13919. Sample 9MFB, 451 m, -49.2506689, -59.1136986, coll. 16/04/2012, ind. 3, NHM.2018.13926–13928. Sample 11MFA, 460 m, -49.2502342, -59.0587417, coll. 15/04/2012, ind. 4, NHM.2018.13968–13971. Sample 17MFB, 450 m, -49.2679625, -59.0563564, coll. 15/04/2012, ind. 4, NHM.2018.14016–14019. Sample 21MFB, 445 m, -49.2866453, -59.1130539, coll. 16/04/2012, ind. 4, NHM.2018.14052–14055. Sample 35MFA, 450 m, -49.3221858, -59.0573711, coll. 15/04/2012, ind. 4, NHM.2018.14208–14211. Sample 45MFB, 434 m, -49.3583828, -59.0842219, coll. 23/04/2012, ind. 4, NHM.2018.14360–14363. Sample 54MFC, 426 m, -49.3943581, -59.0835533, coll. 25/04/2012, ind. 4, NHM.2018.14452–14455. Sample 56MFB, 450 m, -49.2451170, -59.0544011, coll. 18/03/2012, ind. 4, NHM.2018.14480–14483. Sample 56MFC, 450 m, -49.2451170, Longitude -59.0544011, coll. 18/03/2012, ind. 4, NHM.2018.14484–14487. Sample 59MFB, 448 m, -49.2545600, -59.0688839, coll. 19/03/2012, ind. 4, NHM.2018.14516–14519. Sample 66MFA, 445 m, -49.2583220, -59.1058241, coll.18/03/2012, ind. 4, NHM.2018.14596–14599. Sample 70MFC, 446 m, -49.2985060, -59.1150831, coll. 22/03/2012, ind. 4, NHM.2018.14684–14687. Sample 73MFB, 436 m, -49.3416430, -59.1309949, coll. 22/03/2012, ind. 4, NHM.2018.14708–14711. Sample 74MFB, 438 m,-49.3385320, -59.1456000, coll. 22/03/2012, ind. 4, NHM.2018.14744–14747. Sample 79MFA, 446 m, -49.2488410, -59.1867771, coll. 17/03/2012, ind. 4, NHM.2018.14816–14819. Sample 82MFB, 443 m, -49.2614970, -59.1672448, coll. 16/03/2012, ind. 4, NHM.2018.14860–14863. Sample 18MFA, 460 m, -49.2679947, -59.0309117, coll. 24/04/2012, ind. 5, NHM.2018.14954–14958. Sample 36MFA, 452 m, -49.3219581, -59.0298531, coll. 25/04/2012, ind. 5, NHM.2018.15074–15078. Sample 51MFC, 442 m, -49.3949839, -59.1662306, coll. 25/03/2012, ind. 5, NHM.2018.15239–15243. Sample 54MFB, 426 m, -49.3943581, -59.0835533, coll. 25/04/2012, ind. 5, NHM.2018.15259–15263. Sample 59MFC, 448 m, -49.2545600, -59.0688839, coll. 19/03/2012, ind. 5, NHM.2018.15299–15303. Sample 65MFA, 446 m, -49.2584430, -59.1251589, coll. 18/03/2012, ind. 5, NHM.2018.15354–15358. Sample 65MFB, 446 m, -49.2584430, -59.1251589, coll. 18/03/2012, ind. 5, NHM.2018.15364–15368. Sample 90MFB, 440 m, -49.3626660, -59.1204879, coll. 25/03/2012, ind. 5, NHM.2018.15589–15593. Sample 36MFB, 450 m, -49.3219581, -59.0298531, coll. 25/04/2012, ind. 6, NHM.2018.15797–15802. Sample 46MFA, 434 m, -49.3581611, -59.0566836, coll. 14/04/2012, ind. 6, NHM.2018.15839–15844. Sample 58MFB, 453 m, -49.2417960, -59.0496559, coll. 18/03/2012, ind. 6, NHM.2018.15923–15928. Sample 75MFA, 438 m, -49.3383880, -59.1262342, coll. 22/03/2012, ind. 6, NHM.2018.16121–16126. Sample 77MFC, 432 m, -49.3511630, -59.1260079, coll. 22/03/2012, ind. 6, NHM.2018.16139–16144. Sample 16MFC, 459 m, -49.2684428, -59.0858878, coll. 25/04/2012, ind. 7, NHM.2018.16226–16232. Sample 22MFA, 454 m, -49.2864308, -59.0855553, coll. 23/04/2012, ind. 7, NHM.2018.16261–16267. Sample 22MFC, 454 m, -49.2864308, -59.0855553, coll. 23/04/2012, ind. 7, NHM.2018.16275–16281. Sample 28MFB, 451 m, -49.3044189, -59.0852225, coll. 23/04/2012, ind. 7, NHM.2018.16303–16309. Sample 28MFC, 451 m, -49.3044189, -59.0852225, coll. 23/04/2012, ind. 7, NHM.2018.16310–16316. Sample 2MFB, 456 m, -49.2328881, -59.1414894, coll. 24/04/2012, ind. 7, NHM.2018.16317–16323. Sample 55MFB, 449 m, -49.2452600, -59.0641536, coll. 18/03/2012, ind. 7, NHM.2018.16520–16526. Sample 55MFC, 449 m, -49.2452600, -59.0641536, coll. 18/03/2012, ind. 7, NHM.2018.16527–16533. Sample 44MFA, 432 m, -49.3585975, -59.1117606, coll. 14/04/2012, ind. 8, NHM.2018.16984–16991. Sample 73MFC, 436 m, -49.3416430, -59.1309949, coll. 22/03/2012, ind. 8, NHM.2018.17136–17143. Sample 18MFC, 460, -49.2679947, -59.0309117, coll. 24/04/2012, ind. 10, NHM.2018.17581–17590.

##### Description.

Holotype (NHM.2018.21756) incomplete, 9 mm long and 0.7 mm wide for 39 chaetigers; paratypes (NHM.2018.21716 and NHM.2018.14528-31), all incomplete specimens, 6–9 mm long and 0.6–0.8 mm wide for 36–45 chaetigers; body cylindrical (Figs 16a; 19a), narrowing posteriorly. Colour in alcohol pale yellow (Fig. 19a).

Prostomium short, conical, anteriorly pointed (Fig. 16b). Peristomium broad, achaetous, anteriorly bearing a pair of small nuchal organs; eyes absent.

Thorax with 9 chaetigers; notopodial and neuropodial postchaetal lobes very short, knob-like in chaetiger 1–2; notochaetal postchaetal lobes on chaetigers 3–9 initially digitate then becoming long and slender (Figs 16e; 17a); neuropodial postchaetal lobeson chaetigers 3–9 similar to notopodial lobes, but approaching mammiform shape in chaetigers 7–9; no subpodal or ventral papillae.

Abdomen from chaetiger 10 (Fig. 16e); abdominal notopodial postchaetal lobes resemble those of thorax but thinner and longer; abdominal neuropodial postchaetal lobes robust, cylindrical, distally bilobed with both lobes short and blunt, but outer lobe narrower (Figs 16c, d; 17b, d); subpodal flange moderately developed (Figs 16c; 17b); subpodal papillae and interramal cirri absent.

Branchiae from chaetiger 17 or 18 (mostly 18) (Fig. 16f), initially small and triangular, increasing progressively in size, becoming large, strap-like, asymmetrical with distal tip blunt and curved medially, in posterior chaetigers assuming “spear-head like” shape (Figs 16d, g; 17d); posterior branchiae longest, up to twice length of notopodial postchaetal lobes.

All thoracic chaetae camerated capillaries (Fig. 18a, b); in thoracic notopodia few chaetae present (10–20, size dependent), arranged in a single row; in thoracic neuropodia chaetae numerous, arranged in two rows. Abdominal notochaetae include camerated capillaries (Fig. 18b) and forked chaetae, with unequal tynes (Fig. 18c, d), two per fascicle observed; abdominal neurochaetae as long, weakly camerated capillaries only (Fig. 18e). Pygidium not observed.

##### Remarks.

Currently, twelve species of *Leitoscoloplos* are known from the Magellanic province and the Southern Ocean (see Table 2) thanks to revisionary work of Mackie (1987) and more recently Blake (2017). The genus *Leitoscoloplos* became species rich in the southern waters, as previous reports of *L. kerguelensis* (Fig. 19b) were re-examined.

*Leitoscoloplos olei* sp. nov. is very similar to *L. plataensis* Blake, 2017 recorded off Uruguay and Argentina, in the intertidal to 144 m depths in coarse sediments. Both species are small and slender, sharing similar form of prostomium, thoracicand abdominal postchaetal lobes (both noto- and neuropodial) and having asymmetrical branchiae, which Blake (2017) describes as having lateral lobe. The main difference is the start of branchiae, which is in chaetigers 13–16 in *L. plataensis*, but 17–18 (mostly 18) in *L. olei* sp. nov. from Falkland Islands. Additional differences are more subtle. Blake (2017) commented that furcate chaetae are only rarely found in *L. plataensis*, but these are commonly encountered in abdominal notopodia of *L. olei* sp. nov., numbering two per fascicle. The two species also differ in bathymetric distribution, which is shallower in *L. plataensis* (intertidal to 144 m), but deeper in *L. olei* sp. nov. (ca. 450 m).

For full comparison of *Leitoscoloplos* species known from the area see Table 2, as well as discussion in Mackie (1987) and Blake (2017).

**Table 2.** Comparison of *Leitoscoloplos olei* sp. nov. with species known from the region based on literature (Mackie 1987, Blake 2017). An asterisk indicates that only the diagnosis given by Mackie (1987) is considered valid here.

Species	Prostomium	Thorax (no. of chaetigers)	Branchiae (from chaetiger)	Thoracic neuropodial postchaetal lobes (form)	Abdominal notopodial postchaetal lobes (form)	Subpodial flange
L. kerguelensis*	conical, anteriorly slightly rounded	9–10	13–14 (15)	triangular	slender, digitate	rudimentary
***L. abranchiatus***	conical, narrowly rounded	9–13	absent	short, conical	short, digitate	absent? (not reported)
***L. mawsoni***	conical, anteriorly rounded	10	11–12, symmetrical	robust, triangular, digitate	robust, triangular to digitate	initially rudimentary, enlarged blister-like in posterior chaetigers
***L. geminus***	conical, anteriorly rounded	10	11–12, distinctly asymmetrical in posterior chaetigers	approaching oval shape	wide basally, tapering to elongate narrow tip	Initially rudi-mentary, sepa-rated from neuropodium by a distinct notch; enlarged blister-like flanges in posterior chaetigers
L. banzareae*	conical, anteriorly slightly rounded	10	14–15	slender, triangular, digitate*	slender, digitate- lanceolate	narrow
***L. chilensis***	conical, anteriorly sharp	13–16	13–16	mammiform- triangular	foliaceous	well- developed
***L. drakei***	conical, narrowing anteriorly	8–9	18–20	short, stubby, indistinct	elongated and cirriform to narrower and digitiform	absent? (not reported)
***L. eltaninae***	conical, anteriorly sharp	10	20	tapering digitiform	becoming bilobed	absent? (not reported)
***L. nasus***	elongate, more than twice as long as wide; anteriorly narrowly rounded	9–10	13–14	triangular	elongate, becoming narrow more posteriorly	present
***L. phyllobran-chus***	reduced, wider than long, weakly pointed anteriorly	8	17	elliptical	thin, cirriform	neuropodia of middle and posterior segments dorsoventrally swollen, forming weakly developed ventral flange
***L. plataensis***	short, conical, pointed, and narrow	9–10	13–16, asymmetrical, with lateral lobe	mammiform	thin, narrow, digitate	small but distinct, continuous with neuropodial lobe
***L. rankini***	conical, anteriorly pointed	9–11	from anterior abdominal segments	arising from low postchaetal ridge	narrow, digitiform	reported as absent
***L. olei* sp. nov.**	conical, anteriorly pointed	9	17–18, asymmetrical, with ventral lobe	conical to approaching mammiform in posterior thorax	slender, digitate	distinct, continuous with neuropodial lobe

##### Etymology.

Specific name *olei* is genitive case of oleum, which is Latin for oil, to acknowledge the discovery of this species during the oil exploration activities.

##### Distribution.

This species is only known from its type locality, North Falklands Basin, at ca. 450 m depth.

### Cirratulidae Ryckholt, 1851

#### Aphelochaeta longisetosa

Taxon classificationAnimaliaPhyllodocidaPolynoidae

(Hartmann-Schroder, 1965)

3DC707A1-B2FB-5CA4-AE1D-3924BC01CF0F

Figure 20

Tharyx longisetosa Hartmann-Schröder, 1965: 222–223; Carrasco 1977a: 81–82, figs 32–33; Rozbaczylo 1985: 155.
Aphelochaeta longisetosa : Blake 2018: 29–30, fig. 13.

##### Material.

***Holotype***: Off central Chile; 84 m; 39°58'S, 73°44.8'W; coll. 15/03/1960; ZMH P-15068.

##### Description.

**Figure 20.**
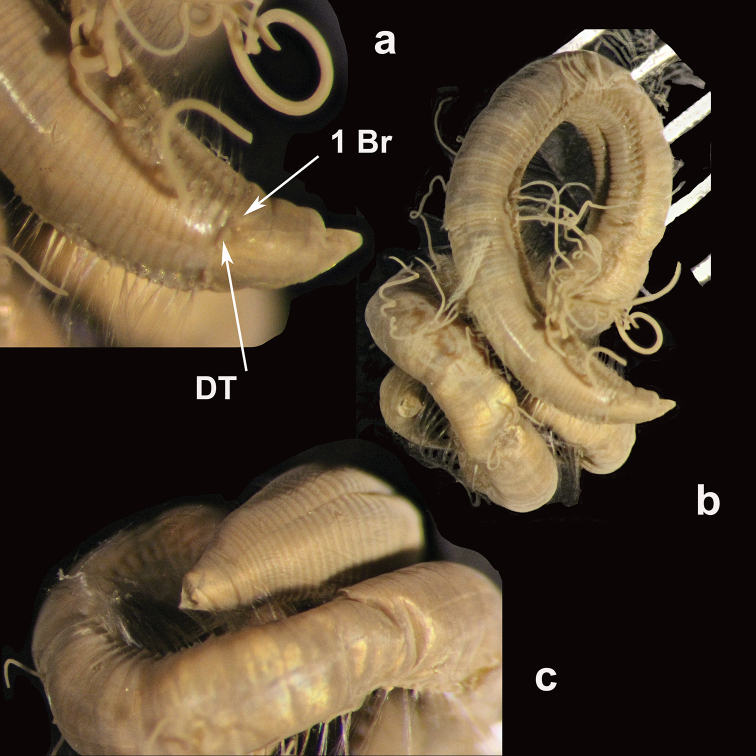
*Aphelochaeta longisetosa* (Hartmann-Schröder, 1965) Holotype (ZMH- P15068) **a** detail of anterior region, showing arrangement of dorsal tentacles (DT) and first branchiae (1 Br) **b** general view of complete specimen **c** view of pygidium, showing pointed anal lobe.

Holotype (ZMH P-15068) complete specimen, olive-brown colour. Body nearly cylindrical with a flattened ventral surface, widening gradually in thorax region, but then the same width to pygidial region; thoracic chaetigers crowded, narrow, wider than long, becoming longer in abdominal chaetigers and again becoming wider than long towards pygidium; no obvious junction between thorax and abdomen. Ventral groove present from mid thorax along body (Fig. 20b, c.)

Prostomium conical, bluntly pointed. Peristomium wider than long, with dorsal crest, three annulations seen laterally and horizontally. Dorsal tentacles separated, situated laterally on distal edge of peristomium at junction with first chaetiger, dorsal and proximal to first branchia. Following branchiae situated dorsal to notochaetae separated by at least one branchial diameter from dorsalmost notochaetae (Fig. 20a).

Chaetae positioned laterally in thoracic segments but becoming more ventral in subsequent segments. Thoracic chaetae relatively long thin capillaries; notochaetae of two sizes, long capillaries of approximately up to ten chaetigers in length, giving specimens a ‘hairy’ appearance, long chaetae appear from anterior thoracic chaetigers and occur down body, only becoming shorter in distal part of abdomen, shorter capillaries less than six chaetigers long also present; thoracic neurochaetae slightly shorter. Pygidial segments widening before narrowing and tapering towards anus, distinct ventral groove observed, terminal anus with low lobes, ventral lobe large extending with an acute tip (Fig. 20c).

##### Methyl Green stain pattern.

Holotype without any discernible pattern.

##### Remarks.

*Aphelochaeta longisetosa* has been only recently redescribed by Blake (2018) based on re- examination of holotype and additional material from offEcuador and Chile. However, as the holotype was never illustrated, we take the opportunity to further update the description of this species by providing images of the holotype (Fig. 20). Few minor differences have been detected between re-description provided here and that of Blake (2018) regarding the position of dorsal tentacles and first pair of branchiae. While Blake (2018) considered these structures to be associated with chaetiger 1, here we observed the dorsal tentacles on distal edge of peristomium and first chaetiger, dorsal and proximal to first branchia. Table 3 gives a comparison of *Aphelochaeta* species that might be found in the Falkland Islands region.

**Table 3.** Table comparing species of *Aphelochaeta* likely to be found in the Falkland Islands region.

Species	Prostomium shape	DT – 1^st^ Br position	Dorsal crest (DC)	Anterior body shape	Ventral groove	MGS
***A. longisetosa***	Conical with rounded blunt tip	DT situated laterally on distal edge of Per; 1^st^ Br lateral to DT	Present on Per area but weakly developed	Widens gradually but then remains same width down to pygidial area; dorsally flat	From mid- thorax extending down abdominal section	No staining pattern observed
**A. cf. longisetosa**	Conical with a blunt- pointed tip	DT separated, situated laterally on distal edge of Peri; 1^st^ Br lateral and distal to DT	Present on Per area	Widens gradual in thoracic region then narrows slightly near junction with abdominal region; dorsally humped	From mid- thorax extending down abdominal section	Band or spot on ventral prostomial region extending into mouth, diffuse staining on sides of peristomium, distinct partial bands ventrally on thoracic chaetigers
***A. falklandica* sp. nov.**	Prostomium wide, conical with rounded or ‘stumpy’ point	DT situated on distal edge of Per, 1^st^ Br lateral or slight posterior.	Present but better developed on proximal part of Per	Body widening gradually in thorax then narrowing at junction with abdomen; dorsally flattened; dorsally flat	From mid to posterior thoracic chaetigers down body	Band ventral under the prostomium extending into the mouth, stain on the lateral part of the peristomium near to the junction with the first chaetiger, narrow bands along lateral and ventral edges of thoracic chaetigers
***A. palmeri***	Blunt pointed with slight indentations at junction with Per	DT arise from junction of Per andCh1; 1^st^ Br lateral and close to DT	Present, well developed extending over all Per	Gradually widening but not narrowing appreciably in anterior part of body	Shallow along entire body	Only weakly/diffuse stain on Per, distinct segmental bands which continue down whole body in some specimens
***A. malefica***	Prostomium pointed, triangular	DT arise from junction of Per with Ch1; 1^st^ Br lateral or slightly anterior to DT arising from junction.	Present, well developed over the distal part of Per	Body not widening appreciably	Absent	No obvious patterns, diffuse staining on prostomium and peristomial region

Key: Ch1= chaetiger 1; DT = dorsal tentacles; Per = peristomium; 1^st^ Br. = first branchia; MGS = methyl green stain.

#### Aphelochaeta cf. longisetosa

Taxon classificationAnimaliaPhyllodocidaPolynoidae

(Hartmann-Schröder, 1965)

B3E80ED4-A6C2-52BF-B68F-D8422B8793C3

**Figure 21.**
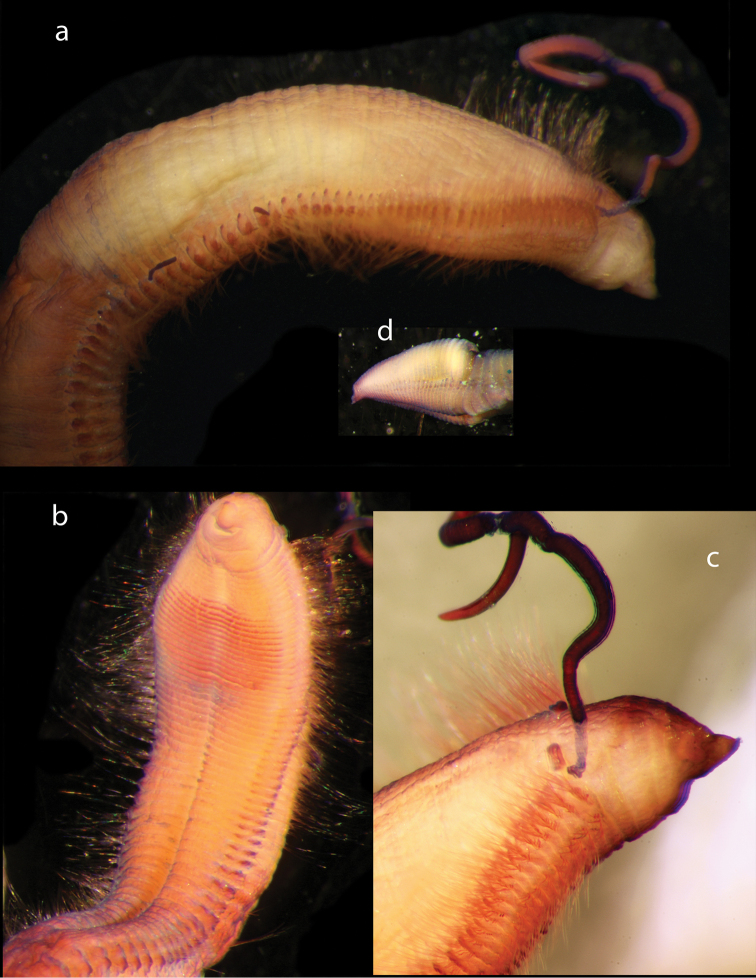
Aphelochaeta cf. longisetosa Falkland Islands specimen **a** dorso-lateral view of anterior of specimen **b** ventral view of anterior **c** close-up showing arrangement of dorsal tentacles and first branchiae **d** pygidium.

**Figure 22.**
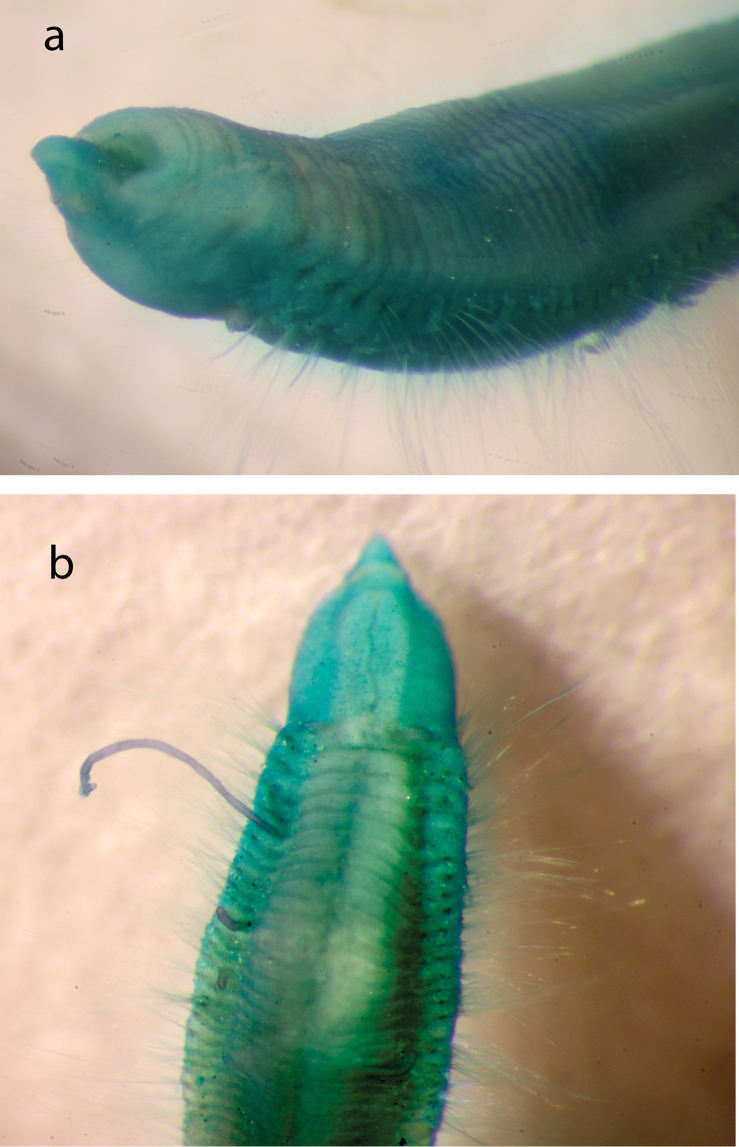
Aphelochaeta cf. longisetosa Falkland Islands specimen – MGSP plate **a** ventrolateral view of anterior end **b** dorsal view of anterior end.

Figures 21 , 22 , 24

##### Materials.

Sample 16MFC, 459 m, -49.268443, -59.085888, coll. 25/04/2012, ind. 1, NHM.2018.21707; Sample 60MFA, 450 m, -49.254527, -59.049453, coll. 18/03/2012, ind. 1, NHM.2018.21754; Sample 20MFB, 451 m, -49.286853, -59.140553, coll. 23/04/2012, ind. 2, NHM.2018.12702–12703.

##### Description.

Specimen 16 MFC anterior fragment 16.3mm long with 79 chaetigers, specimen 60 MFA 11mm long with 67 chaetigers. Body widening gradually in thorax region, dorsally hump-backed, thoracic chaetigers crowded, narrow, wider than long, becoming longer and more flaccid in abdominal chaetigers; ventrally, groove present from mid thorax down the body (Fig. 21b).

Prostomium conical, bluntly pointed. Peristomium wider than long, domed dorsal crest over first two annulations of peristomium, three annulations seen laterally and horizontally (Fig. 21a, c). Dorsal tentacles separated, situated laterally on distal edge of peristomium and first chaetiger, dorsal and proximal to first branchiae (Fig. 21c). Following branchiae situated dorsal to notochaetae separated by at least one branchial diameter from dorsalmost notochaetae.

Chaetae positioned laterally in thoracic segments. Thoracic notochaetae of two sizes, long capillaries approximately up to 10 chaetigers long, giving specimens a‘silky’ appearance, long chaetae similar in length perhaps slightly longer in distalmost thoracic chaetigers; thoracic neurochaetae slightly shorter; 7–10 notochaetae and 6–8 neurochaetae per fascicle. Abdominal notochaetae fewer in number than neurochaetae and less dense than in thoracic chaetigers; a few long chaetae appear from anterior thoracic chaetigers and occur down body into the abdominal chaetigers, shorter capillaries less than six chaetigers in length also present.

Pygidial region expanded, pygidium with lobes the ventralmost enlarged and pointed (Figs 21d; 24a)

##### Methyl Green stain pattern.

Falkland Islands specimens with band or spot on ventral prostomial region extending into mouth, diffuse staining on sides of peristomium, distinct partial bands ventrally on thoracic chaetigers along edges of the chaetigers, becoming denser in mid and proximal thoracic chaetigers, some banding dorsally on thoracic chaetigers (Fig. 22).

##### Remarks.

Aphelochaeta cf. longisetosa (Hartmann-Schröder, 1965) was identified from a number of samples during the contract, but only a few specimens have so far been registered in the Museum’s collection. The Falkland Islands specimens closely resemble Hartmann-Schröder’s holotype, particularly in possessing the long silky chaetae (see Table 3). The Falkland Islands specimens differ in the shape of the body; they show a distinct staining pattern which is absent in *A. longisetosa*; however, this might be due to difference in the age of the specimens, and the abdominal notochaetae are fewer in number than neurochaetae and less dense than in thoracic chaetigers. Given these observations and that the Falkland Islands lie outside the previous range of *A. longisetosa* we are taking a cautious position and assigning the specimens to A. cf. longisetosa.

Aphelochaeta cf. longisetosa resembles *A. falklandica* sp. nov. having similar MGSP but differs in the shape of the body, A. cf. longisetosa has a near cylindrical body with only a slight widening in the proximal thoracic region and with no obvious junction between the thorax and abdomen, whereas in *A. falklandica* sp. nov. the thorax is flat with the body widening in the thoracic region and narrowing again at the junction between the thorax and abdomen; finally, the pygidium has an extended lobe with a pointed tip, whereas *A. falklandica* sp. nov. has a rounded ventral lobe. Such differences appear to be consistent and so it has been decided to consider them as separate species.

In terms of other known *Aphelochaeta* species in the region, A. cf. longisetosa resembles *A. malefica* Elias & Rivero, 2009, in the body shape as the species from the River Plate has a body more uniform in width. However, the two species differ in that the peristomium is much shorter in the Falkland Islands specimens, only as long as wide, whereas in *A. malefica* Elias & Rivero, 2009 the peristomium is longer than wide in the position of the dorsal tentacles and first branchiae and that the latter also has the peristomium extending back displacing the first chaetiger, while A. cf. longisetosa the junction is not displaced proximally. The MGSP for the two species is also different. The Falkland Islands species have only diffuse staining in the peristomium and prostomium while *A. malefica* has these areas darkly stained.

##### Distribution.

NW Falkland Islands at depths of 400–500 m.

#### Aphelochaeta falklandica

Taxon classificationAnimaliaPhyllodocidaPolynoidae

Paterson & Neal sp. nov.

6E46C0CB-2BB7-585D-97D6-1226712CEB93

http://zoobank.org/7314F6D0-4EEB-4C45-9F02-0251A55044E2

Figures 23 , 24

##### Materials.

***Holotype***: Sample 17MFA, 449 m, -49.2679625, -59.0563564, coll. 15/04/2012, ind. 1, NHM.2018.21708. ***Paratypes***: Sample 31MFA, 442 m, -49.3230311, -59.1674461, coll. 15/04/2012, ind. 1, NHM.2018.21728. Sample 44MFB, 429 m, -49.3585975, -59.1117606, coll. 14/04/2012, ind. 1, NHM.2018.21744. Sample 6MFC, 458 m, -49.2320192, -59.0316156, coll. 25/04/2012, ind. 2, NHM.2018.12714–12715. Sample 66MFA, 445 m, -49.258322, -59.1058241, coll. 18/03/2012, ind. 1, NHM 2018.25373. Sample T10MFB, 702 m, -53.059303, -58.026135, coll. 02/02/2009, ind. 1, NHM.2018.25370.

##### Description.

Holotype NHM.2018.21708, anterior fragment 14.1mm in length with 127 chaetigers. Body widening gradually in thorax then narrowing at junction with abdomen; dorsally flattened; ventrally a shallow groove runs down body from mid to posterior thorax. Thoracic chaetigers much wider than long, crowded; in abdomen chaetigers, as wide as long or longer, before becoming crowded in the pygidial segments.

**Figure 23.**
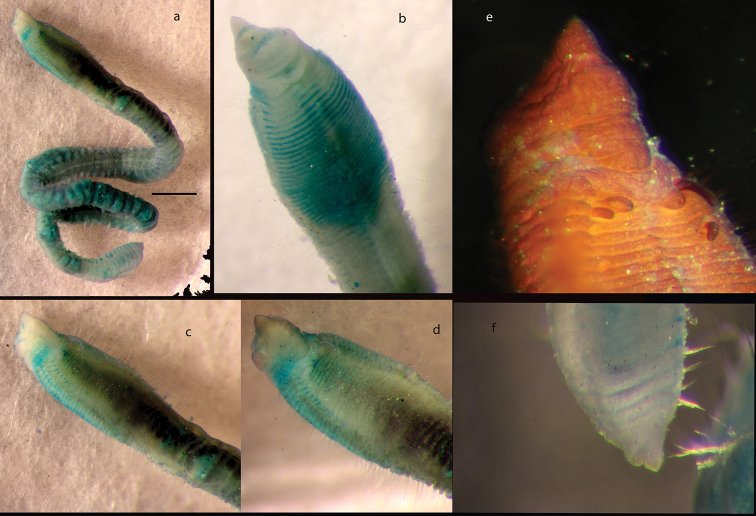
*Aphelochaeta falklandica* sp. nov. (holotype NHM.2018.21708), Methyl Green Staining patterns **a** complete specimen **b** ventral view of thoracic chaetigers showing methyl green stained bands across the thorax **c** dorsal view of head and thoracic chaetigers **d** close up of head region **e** dorsal view of anterior of specimen stained with Shirla stain showing dorsal tentacles **f** detail of pygidial region showing anal lobes. Scale bars: 1 mm (**a**).

**Figure 24.**
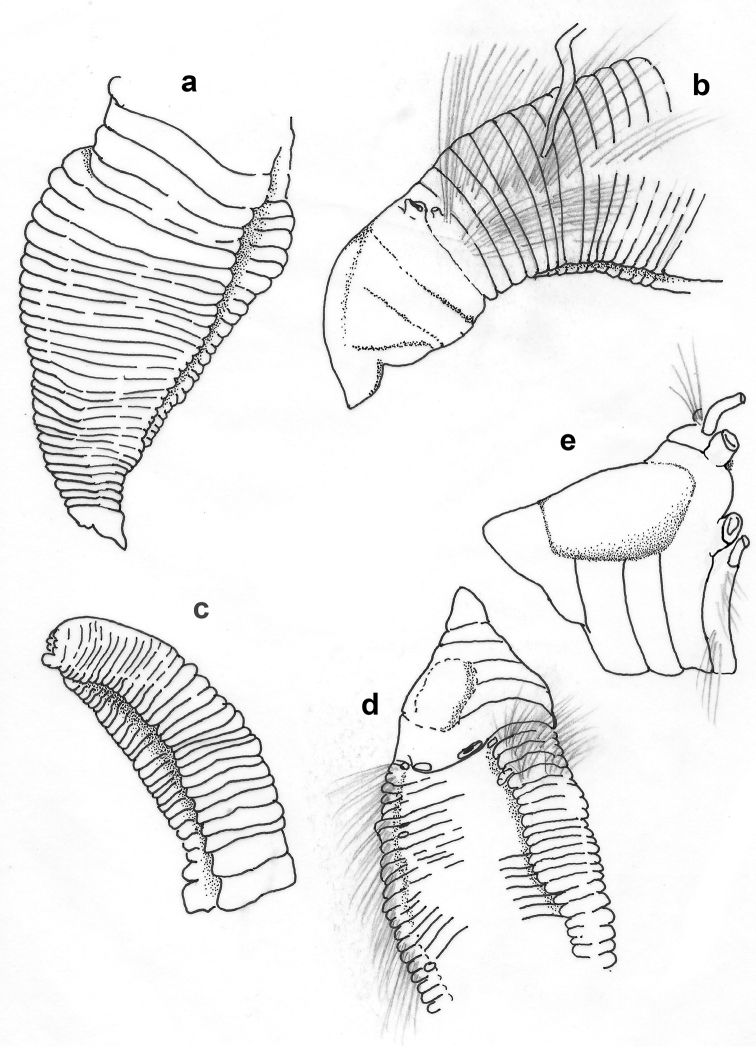
Comparison of *Aphelochaeta falklandica* sp. nov. and A. cf. longisetosa (Hartmann-Schröder, 1965). Aphelochaeta cf. longisetosa**a** drawing of pygidium showing pointed ventral anal lobe **b** drawing of anterior showing rounded peristomium. *Aphelochaeta falklandica* sp. nov. **c** drawing of pygidium showing rounded ventral anal lobe **d** drawing of anterior showing domed dorsal peristomium and widened flat thoracic chaetigers **e** detail drawing of prostomium and peristomial area showing arrangement of dorsal tentacles and 1^st^ branchiae.

Prostomium wide, conical with rounded or ‘stumpy’ point. Peristomium as wide as long, with domed dorsal crest; three annulations, first two obvious in lateral and ventral view, interrupted by dorsal crest, third complete dorsally and extending distally into first chaetiger. Dorsal tentacles arising between peristomium and first chaetiger, sometimes appearing to arise in the first chaetiger due to the peristomium extending back into the first chaetiger; dorsal to and on the same level or slightly anterior to first branchiae; second branchial pair close to first pair (Figs 23e; 24e).

Chaetae all simple capillaries, thoracic notochaetae of two basic lengths-relatively short capillaries ca. four chaetigers in length or equivalent to half the chaetiger width, and longer chaetae of at least ten chaetigers length; long chaetae arising from posterior thoracic region occurring in subsequent chaetigers into abdominal region; 6–10 chaetae per fascicle sometime appearing as if in two groups. Thoracic neurochaetae short capillaries, similar in length as short capillaries in the notopodia; 5–6 chaetae per fascicle. Abdominal notochaetae longer than those of neuropodia, smaller numbers of chaetae in abdominal fascicles than in thorax.

Pygidium with last 18 segments expanded, anal opening with lobes, ventralmost lobe largest, blunt and extended (Figs 23f; 24c).

##### Methyl Green stain pattern.

Band ventral under the prostomium extending into the mouth, narrow bands along lateral and ventral edges of thoracic chaetigers (Fig. 23).

##### Remarks.

*Aphelochaeta falklandica* sp. nov. is a common species in the Sealion samples. It closely resembles A. cf. longisetosa (Hartmann-Schröder 1965), differing in being more dorso-ventrally flattened with an obvious widening of the body in the thoracic region, with an obvious junction between the thoracic and abdominal chaetigers and in the blunt shape of the ventral anal lobe (see Figs 23f; 24c). In mixed samples thisspecies can often be distinguished by a full gut obvious in posterior thoracic and anterior abdominal chaetigers and, in fresh material, by the bases of branchiae in the posterior thorax showing as bright red dots. The domed dorsal crest is also characteristic.

*Aphelochaeta falklandica* sp. nov. closely resembles *A. palmeri* Blake, 2018 and *A. spectabilis* Blake, 2018 both recently described from Antarctic and South American waters; *A. falklandica* sp. nov. has a similar body shape to these species but appears to differ in the following ways: the definition of the annulation differs slightly in that with *A. spectabilis* the junctions of the annulations are only weakly defined whereas in *A. falklandica* sp. nov. the junctions of the annulations are distinct although can be less easy to see dorsally if the dorsal crest is well developed. The dorsal tentacles of *A. spectabilis* arise on the posterior part of the peristomium or in the junction between the peristomium and first chaetiger, with the first branchia arising on the first chaetiger. In *A. falklandica* sp. nov. the dorsal tentacle also arises in the junction between the first chaetiger and peristomium but the first branchia arises ventral to the dorsal tentacle and in the junction between the first chaetiger and the peristomium. *Aphelochaeta falklandica* sp. nov. has a ventral groove running from the mid to posterior thoracic region of the body. *Aphelochaeta spectabilis* does not have a ventral groove. In *A. spectabilis* thedevelopment and shape of the posterior chaetigers and pygidial region is fusiform and well developed with a simple pygidium with a rounded lobe, whereas in *A. falklandica* sp. nov. this region is not as fusiform and the pygidium has an extended blunt ventral lobe. The staining pattern for *A. spectabilis* differs from *A. falklandica*, being more developed occurring dorsally on the peristomium, there is a distinct patch posterior of the mouth and the first chaetiger. These patches are missing from *A. falklandica* sp. nov., but this species has patches on the lateral parts of the peristomium, there is a small band ventrally on the prostomium.

The shape of the prostomium in *A. palmeri* is pear-shaped, slightly indented at its junction with the peristomium; the prostomium in *A. falklandica* sp. nov. is conical and with a blunt rounded tip. The dorsal crest in *A. falklandica* is not always well developed and may extend only as far as the second annulation whereas the dorsal crest in *A. palmeri* is usually well developed extending to the end of the peristomium. Aphelochaeta*palmeri* has a well-developed posterior region with a fusiform shape and a blunt lobed pygidium. In *A. falklandica* sp. nov. this region is not so well developed and expanded. The staining pattern differs in that there are distinct bands ventrally in the prostomium and a large patch laterally on each side of the peristomium in *A. falklandica* sp. nov. which are absent in *A. palmeri*.

*Aphelochaeta falklandica* sp. nov. can be distinguished from the *A. malefica* Elias & Rivero, 2009 by the arrangement of the dorsal tentacles, third annulation of the peristomium and MGSP. The shape of the body suggests similarities with *A. williamsae* Blake, 1996 from California but *A. falklandica* sp. nov. differs in MGSP which have banding extending laterally while in *A. williamsae* they do not. *Aphelochaeta falklandica* sp. nov. differs from the other Californian species in that the dorsal thorax is flattened and ventrally more rounded, whereas most of Blake’s (1996) species are dorsal-ventrally rounded or have a rounded (hump-backed) dorsal thoracic shape. Table 3 gives the comparison of *Aphelochaeta* species that might be found in the Falkland Islands region.

##### Etymology.

This species is named after the Falkland Islands where it was discovered.

##### Distribution.

Recorded from the NW Falkland Islands at depths of 400–500 m

#### Dodecaceria saeria

Taxon classificationAnimaliaPhyllodocidaPolynoidae

Paterson & Neal sp. nov.

73C92185-12A7-54AC-AFA0-89EC218B410E

http://zoobank.org/2B2358A7-ACF7-437A-90BA-B27DB038E436

Figures 25 , , 27

##### Material.

***Holotype***: Sample T5MFB, 610 m, -53.028677, -59.067642, coll. 02/02/2009, ind. 1, NHM.2018.23622. ***Paratypes***: Sample 55MFB, 449 m, -49.24526, -59.064154, coll. 18/03/2012, ind. 1, NHM.2018.25370. Sample 3MFC, 464 m, -49.232681, -59.114021, coll. 16/04/2012, ind. 1, NHM.2018.25371.

##### Description.

**Figure 25.**
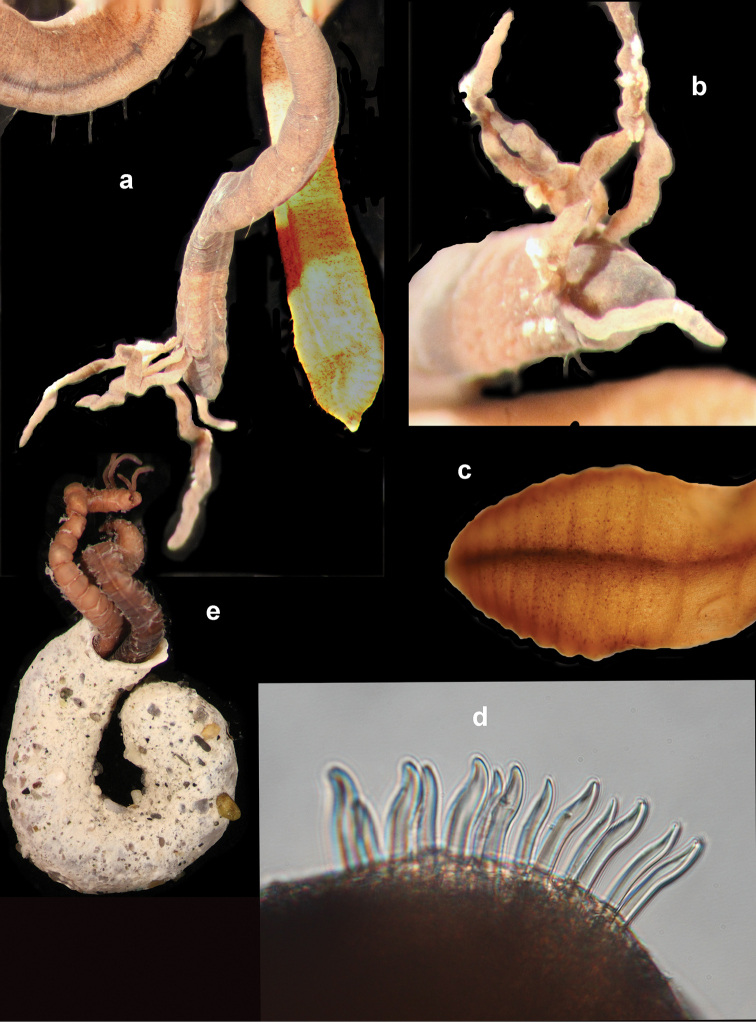
*Dodecaceria saeria* sp. nov. (holotype NHM.2018.23622) **a** general views of anterior and posterior ends of worm **b** close-up of anterior **c** pygidium **d** chaetae of mid-body chaetiger **e** tube.

Holotype NHM.2018.23622, anterior body brown in colour becoming lighter down body; cylindrical or slightly dorso-ventrally flattened indistal segments around pygidium in particular; paratype NHM.2018.25371, 16 mm in length with 90 segments. Ventral groove present in abdominal segments (Fig. 25c).

**Figure. 26.**
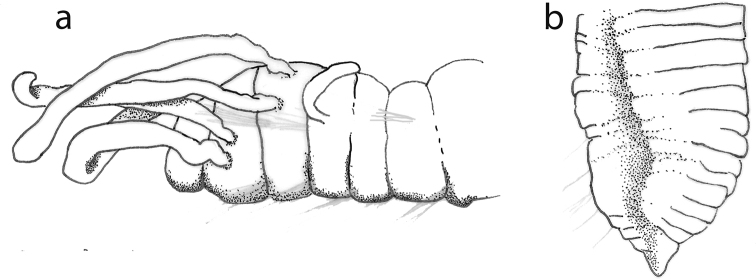
*Dodecaceria saeria* sp. nov. **a** dorso-lateral view of anterior end **b** pygidium and distal segments.

Prostomium trapezoid with a narrow slightly rounded free edge. Peristomium short. Dorsal tentacles arising laterally, long and club-shaped, first pair of branchiae arise dorsal to dorsal tentacles. Two or three pairs branchiae, 2^nd^ pair smaller than first, approximately half to two-thirds the length, 3^rd^ pair same length as 2^nd^ pair (Figs 25a, b; 26a).

No achaetous segment, chaetigerous segments start after peristomium. Anterior chaetigers with simple capillaries, five in notopodia and four in neuropodia. Spines beginning on chaetiger 22–29; podia with mix of capillaries and spines, up to six spines and four to five capillaries, becoming 8–13 spines in fascicules of distal chaetigers. Spines with excavated tips, smooth edges. Chaetigers of pygidial area with only capillaries.

Posterior chaetigers flattened, slightly widened laterally and slightly excavated ventrally, pygidium ventral with large rounded lobe dorsally (Figs 25c; 26b).

Tube blind-ended, hard, calcareous with mineral inclusions; surface smooth (Fig. 25e).

##### Remarks.

**Figure 27.**
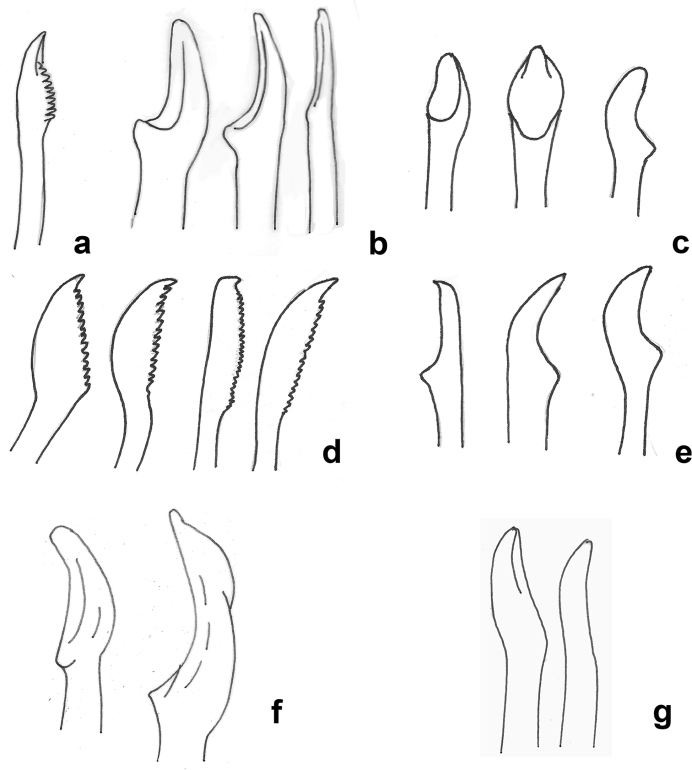
Comparison of spines of *Dodecaceria* species recorded from the region **a***D. gallardoi* Carrasco, 1977 **b***D. choromytilicola* Carrasco, 1977; *D. fistulicola* Ehlers, 1901 **d***D. multifiligera* Hartmann-Schröder, 1962 **e***D. laddi oculata* Hartmann-Schröder, 1962 **f***D. meridiana* Elias & Rivero, 2009 **g***D. saeria* sp. nov.

Seven species of *Dodecaceria* have been recorded in the region (see Table 4 and Fig. 27). Three species also have two or three pairs of branchiae: *D. gallardoi* Carrasco, 1977b from Chile, *D. laddi* Hartman, 1954 from the Marshall Islands, and *D. laddi oculata* Hartmann-Schröder, 1962 from Peru. *Dodecaceria saeria* sp. nov. appears to differ from these species in the shape of the spoon-like spines (Fig. 22) and in the position where these spines first arise, chaetiger 22 to 29 as compared with much earlier in the three other species.

**Table 4.** Comparison of *Dodecaceria* species in the region and those with similar characters to the Falkland Island species.

Species	Branchial pairs	No. of branchial pairs in 1^st^ branchial position	Branchiae monomorphic or dimorphic	Insertion of palps	Spines appearance
***D. choromytilicola***	13	2	Dimorphic	Lateral	Ch11
***D. fistulicola***	5–7	1	Dimorphic	Lateral	No Ch 11–14 Ne Ch 9–12
***D. gallardoi***	3	2	Monomorphic	Lateral	Ch6
***D. meridiana***	7–18	1	Dimorphic	Lateral	Ch8
***D. multifiligera***	22	1	Monomorphic	Dorsal	Ch6
***D. opulens***	7–14	2	Dimorphic	Lateral	Ch8
***D. laddi oculata***	2	?	?	?	Ch3–8
***D. saeria sp. nov.***	2–3	1	Dimorphic	Lateral	Ch22–29

Key: Ch = chaetiger, Ne-neuropodia; No-notopodia.

##### Etymology.

This species is named after the South Atlantic Environmental Research institute (SAERI) which has done so much to support the taxonomic work included in this paper.

##### Distribution.

This species has only been recorded from the Falkland Islands. *Dodecaceria* species are more commonly associated with rock or shell burrowers. The log of the cruise states “Mostly black gravel up to 1.5cm diameter in sieve residue” which may account for the occurrence of this taxon in predominately soft sediment.

## Checklist of polychaete species and morphospecies included in the on-line taxonomic guide

### Family Ampharetidae Malmgren, 1866

Falkland Islands Scratchpad http://falklands.myspecies.info/simpletaxonomy/term/209

***Amage sculpta* Ehlers, 1908**

Falkland Islands Scratchpad http://falklands.myspecies.info/simpletaxonomy/term/211

***Ampharete* sp. 1**

Falkland Islands Scratchpad http://falklands.myspecies.info/simpletaxonomy/term/213

***Amphicteis* sp. 1**

Falkland Islands Scratchpad http://falklands.myspecies.info/simpletaxonomy/term/215

***Amphitritides* sp. 1**

Falkland Islands Scratchpad http://falklands.myspecies.info/simpletaxonomy/term/369

**Anobothrus cf. glandularis (Hartmann-Schröder, 1965)**

Falkland Islands Scratchpad http://falklands.myspecies.info/simpletaxonomy/term/218

***Anobothrus* sp. 1**

Falkland Islands Scratchpad http://falklands.myspecies.info/simpletaxonomy/term/219

***Eclysippe* sp. 1**

Falkland Islands Scratchpad http://falklands.myspecies.info/simpletaxonomy/term/220

***Eusamythella sexdentata* (Hartman, 1967)**

Falkland Islands Scratchpad http://falklands.myspecies.info/simpletaxonomy/term/222

***Glyphanostomum* sp. 1**

Falkland Islands Scratchpad http://falklands.myspecies.info/simpletaxonomy/term/224

**Lysippe cf. fragilis (Wollebaeck, 1912)**

Falkland Islands Scratchpad http://falklands.myspecies.info/simpletaxonomy/term/227

***Lysippe* sp. 1**

Falkland Islands Scratchpad http://falklands.myspecies.info/simpletaxonomy/term/228

***Lysippe* sp. 2**

Falkland Islands Scratchpad http://falklands.myspecies.info/simpletaxonomy/term/229

***Melinna arnaudi* Parapar & San Martín, 1997**

Falkland Islands Scratchpad http://falklands.myspecies.info/simpletaxonomy/term/239

***Melinna* sp. 1**

Falkland Islands Scratchpad http://falklands.myspecies.info/simpletaxonomy/term/240

***Samytha* sp. 1**

Falkland Islands Scratchpad http://falklands.myspecies.info/simpletaxonomy/term/230

***Samythella* sp. 1**

Falkland Islands Scratchpad http://falklands.myspecies.info/simpletaxonomy/term/233

***Samythella* sp. 2**

Falkland Islands Scratchpad http://falklands.myspecies.info/simpletaxonomy/term/234

***Tanseimaruana* sp. 1**

Falkland Islands Scratchpad http://falklands.myspecies.info/simpletaxonomy/term/235

### Family Amphinomidae Lamarck, 1818

Falkland Islands Scratchpad http://falklands.myspecies.info/simpletaxonomy/term/431

***Paramphinome australis* Monro, 1930**

Falkland Islands Scratchpad http://falklands.myspecies.info/simpletaxonomy/term/431

### Family Capitellidae Grube, 1862

Falkland Islands Scratchpad http://falklands.myspecies.info/simpletaxonomy/term/401

***Capitella* sp. 1**

Falkland Islands Scratchpad http://falklands.myspecies.info/simpletaxonomy/term/402

***Capitella* sp. 2**

Falkland Islands Scratchpad http://falklands.myspecies.info/simpletaxonomy/term/403

***Capitella* sp. 3**

Falkland Islands Scratchpad http://falklands.myspecies.info/simpletaxonomy/term/404

***Neomediomastus* sp. 1**

Falkland Islands Scratchpad http://falklands.myspecies.info/simpletaxonomy/term/406

***Notomastus* sp. 1**

Falkland Islands Scratchpad http://falklands.myspecies.info/simpletaxonomy/term/405

### Family Chaetopteridae Audouin & Milne Edwards, 1833

Falkland Islands Scratchpad http://falklands.myspecies.info/simpletaxonomy/term/407

***Phyllochaetopterus* sp. 1**

Falkland Islands Scratchpad http://falklands.myspecies.info/simpletaxonomy/term/409

***Spiochaetopterus* sp. 1**

Falkland Islands Scratchpad http://falklands.myspecies.info/simpletaxonomy/term/408

### Family Cirratulidae Ryckholt, 1851

***Chaetocirratulus andersenensis* (Augner, 1932)**

Falkland Islands Scratchpad http://falklands.myspecies.info/simpletaxonomy/term/453

***Aphelochaeta* sp. 1**

Falkland Islands Scratchpad http://falklands.myspecies.info/simpletaxonomy/term/446

***Caulleriella* sp.**

Falkland Islands Scratchpad http://falklands.myspecies.info/simpletaxonomy/term/454

***Chaetozone* sp. 1**

Falkland Islands Scratchpad http://falklands.myspecies.info/simpletaxonomy/term/439

***Chaetozone* sp. 2**

Falkland Islands Scratchpad http://falklands.myspecies.info/simpletaxonomy/term/440

***Chaetozone* sp. 3**

Falkland Islands Scratchpad http://falklands.myspecies.info/simpletaxonomy/term/441

***Chaetozone* sp. 4**

Falkland Islands Scratchpad http://falklands.myspecies.info/simpletaxonomy/term/442

***Chaetozone* sp. 6**

Falkland Islands Scratchpad http://falklands.myspecies.info/simpletaxonomy/term/443

***Chaetozone* sp. 8**

Falkland Islands Scratchpad http://falklands.myspecies.info/simpletaxonomy/term/444

***Cirratulus* sp. 1**

Falkland Island Scratchpad http://falklands.myspecies.info/simpletaxonomy/term/452

***Dodecaceria* sp. 2**

Falkland Islands Scratchpad http://falklands.myspecies.info/simpletaxonomy/term/438

**Chaetocirratulus cf. epitocus (Monro, 1930)**

Falkland Islands Scratchpad http://falklands.myspecies.info/simpletaxonomy/term/544

### Family Cossuridae Day, 1963

Falkland Islands Scratchpad http://falklands.myspecies.info/simpletaxonomy/term/485

***Cossura* sp. 1**

Falkland Islands Scratchpad http://falklands.myspecies.info/simpletaxonomy/term/485

### Family Dorvilleidae Chamberlin, 1919

Falkland Islands Scratchpad http://falklands.myspecies.info/simpletaxonomy/term/462

***Schistomeringos* sp 1**

Falkland Islands Scratchpad http://falklands.myspecies.info/simpletaxonomy/term/462

### Family Eunicidae Berthold, 1827

Falkland Islands Scratchpad http://falklands.myspecies.info/simpletaxonomy/term/489

**Eunice cf. pennata (Müller, 1776)**

### Family Euphrosinidae Williams, 1852

Falkland Islands Scratchpad http://falklands.myspecies.info/simpletaxonomy/term/429

***Euphrosinopsis antipoda* Kudenov, 1993**

Falkland Islands Scratchpad http://falklands.myspecies.info/simpletaxonomy/term/490

**Euphrosinopsis cf. crassiseta Kudenov, 1993**

Falkland Islands Scratchpad http://falklands.myspecies.info/simpletaxonomy/term/491

### Family Fabriciidae Rioja, 1923

Falkland Islands Scratchpad http://falklands.myspecies.info/simpletaxonomy/term/241

***Fabricinuda* sp. 1**

Falkland Islands Scratchpad http://falklands.myspecies.info/simpletaxonomy/term/243

***Fabricinuda* sp. 2**

Falkland Islands Scratchpad http://falklands.myspecies.info/simpletaxonomy/term/244

**cf. *Novafabricia* sp. 1**

Falkland Islands Scratchpad http://falklands.myspecies.info/simpletaxonomy/term/351

### Family Fauveliopsidae Hartman, 1971

Falkland Islands Scratchpad http://falklands.myspecies.info/simpletaxonomy/term/416

**Laubieriopsis cf. brevis Hartman, 1967**

Falkland Islands Scratchpad http://falklands.myspecies.info/simpletaxonomy/term/416

### Family Flabelligeridae de Saint-Joseph, 1894

Falkland Islands Scratchpad http://falklands.myspecies.info/simpletaxonomy/term/470

***Brada* sp. 1**

Falkland Islands Scratchpad http://falklands.myspecies.info/simpletaxonomy/term/484

### Family Glyceridae Grube, 1850

Falkland Islands Scratchpad http://falklands.myspecies.info/simpletaxonomy/term/415

***Glycera* sp. 1**

Falkland Islands Scratchpad http://falklands.myspecies.info/simpletaxonomy/term/502

***Glycera* sp. 2**

Falkland Islands Scratchpad http://falklands.myspecies.info/simpletaxonomy/term/493

### Family Lacydoniidae Bergström, 1914

Falkland Islands Scratchpad http://falklands.myspecies.info/simpletaxonomy/term/476

***Lacydonia* sp. 1**

### Family Lumbrineridae Schamada, 1861

Falkland Islands Scratchpad http://falklands.myspecies.info/simpletaxonomy/term/455

? ***Eranno* sp. 1**

Falkland Islands Scratchpad http://falklands.myspecies.info/simpletaxonomy/term/458

***Abyssoninoe abyssorum* McIntosh, 1885**

Falkland Islands Scratchpad http://falklands.myspecies.info/simpletaxonomy/term/459

***Augeneria tentaculata* Monro, 1930**

Falkland Islands Scratchpad http://falklands.myspecies.info/simpletaxonomy/term/457

***Lumbrineris kerguelensis/cingulata* group**

Falkland Islands Scratchpad http://falklands.myspecies.info/simpletaxonomy/term/456

***Ninoe falklandica* Monro, 1936**

Falkland Islands Scratchpad http://falklands.myspecies.info/simpletaxonomy/term/460

### Family Maldanidae Malmgren, 1867

Falkland Islands Scratchpad http://falklands.myspecies.info/simpletaxonomy/term/331

***Boguea* sp. 1**

Falkland Islands Scratchpad http://falklands.myspecies.info/simpletaxonomy/term/334

***Clymenura* sp. 1**

Falkland Islands Scratchpad http://falklands.myspecies.info/simpletaxonomy/term/342

***Clymenura* sp. 2**

Falkland Islands Scratchpad http://falklands.myspecies.info/simpletaxonomy/term/341

***Clymenura* sp. 3**

Falkland Islands Scratchpad http://falklands.myspecies.info/simpletaxonomy/term/483

***Euclymeninae* sp. 2**

Falkland Islands Scratchpad http://falklands.myspecies.info/simpletaxonomy/term/354

***Lumbriclymenella* sp. 1**

Falkland Islands Scratchpad http://falklands.myspecies.info/simpletaxonomy/term/338

***Micromaldane* sp. 1 (larva?)**

Falkland Islands Scratchpad http://falklands.myspecies.info/simpletaxonomy/term/335

**Nichomache cf. lumbricalis (Fabricius, 1780)**

Falkland Islands Scratchpad http://falklands.myspecies.info/simpletaxonomy/term/336

***Notoproctus* sp. 1**

Falkland Islands Scratchpad http://falklands.myspecies.info/simpletaxonomy/term/337

***Praxillella* sp. 1**

Falkland Islands Scratchpad http://falklands.myspecies.info/simpletaxonomy/term/495

**Rhodine cf. antarctica Gravier, 1911**

Falkland Islands Scratchpad http://falklands.myspecies.info/simpletaxonomy/term/348

**Rhodine cf. intermedia Arwidsson, 1911**

Falkland Islands Scratchpad http://falklands.myspecies.info/simpletaxonomy/term/347

### Family Nephtyidae Grube, 1850

Falkland Islands Scratchpad http://falklands.myspecies.info/simpletaxonomy/term/322

**Aglaophamus cf. peruana (Hartman, 1940)**

Falkland Islands Scratchpad http://falklands.myspecies.info/simpletaxonomy/term/327

***Aglaophamus posterobranchus* Hartman, 1967**

Falkland Islands Scratchpad http://falklands.myspecies.info/simpletaxonomy/term/326

***Aglaophamus* sp. 1**

Falkland Islands Scratchpad http://falklands.myspecies.info/simpletaxonomy/term/328

***Aglaophamus* sp. 2**

Falkland Islands Scratchpad http://falklands.myspecies.info/simpletaxonomy/term/329

***Aglaophamus* sp. 3**

Falkland Islands Scratchpad http://falklands.myspecies.info/simpletaxonomy/term/330

**Nephtys cf. paradoxa Malm, 1874**

Falkland Islands Scratchpad http://falklands.myspecies.info/simpletaxonomy/term/323

### Family Nereididae Blainville, 1818

Falkland Islands Scratchpad http://falklands.myspecies.info/simpletaxonomy/term/467

***Ceratocephale* sp. 1**

Falkland Islands Scratchpad http://falklands.myspecies.info/simpletaxonomy/term/475

***Ceratocephale* sp. 2**

Falkland Islands Scratchpad http://falklands.myspecies.info/simpletaxonomy/term/482

***Neanthes* sp. 1**

Falkland Islands Scratchpad http://falklands.myspecies.info/simpletaxonomy/term/474

### Family Oenonidae Kinberg, 1865

Falkland Islands Scratchpad http://falklands.myspecies.info/simpletaxonomy/term/464

***Drilonereis* sp. 1**

Falkland Islands Scratchpad http://falklands.myspecies.info/simpletaxonomy/term/486

***Drilonereis tenuis* (Ehlers, 1901)**

Falkland Islands Scratchpad http://falklands.myspecies.info/simpletaxonomy/term/488

### Family Onuphidae Kinberg, 1865

Falkland Islands Scratchpad http://falklands.myspecies.info/simpletaxonomy/term/381

**Anchinothria cf. pycnobranchaiata McIntosh, 1885**

Falkland Islands Scratchpad http://falklands.myspecies.info/simpletaxonomy/term/391

***Kinbergonuphis oligobranchiata* (Orensanz, 1974)**

Falkland Islands Scratchpad http://falklands.myspecies.info/simpletaxonomy/term/389

***Leptoecia vivipara* Orensanz, 1990**

Falkland Islands Scratchpad http://falklands.myspecies.info/simpletaxonomy/term/387

***Nothria anoculata* Orensanz, 1974**

Falkland Islands Scratchpad http://falklands.myspecies.info/simpletaxonomy/term/390

***Onuphis pseudoiridescens* Averincev, 1972**

Falkland Islands Scratchpad http://falklands.myspecies.info/simpletaxonomy/term/388

### Family Opheliidae Malmgren, 1867

Falkland Islands Scratchpad http://falklands.myspecies.info/simpletaxonomy/term/189

**Ophelina cf. abranchiata Støp-Bowitz, 1948**

Falkland Islands Scratchpad http://falklands.myspecies.info/simpletaxonomy/term/193

**Ophelina cf. cylindricaudata (Hansen, 1878)**

Falkland Islands Scratchpad http://falklands.myspecies.info/simpletaxonomy/term/192

***Ophelina scaphigera* (Ehlers, 1900)**

Falkland Islands Scratchpad http://falklands.myspecies.info/simpletaxonomy/term/194

***Ophelina syringopyge* (Ehlers, 1901)**

Falkland Islands Scratchpad http://falklands.myspecies.info/simpletaxonomy/term/195

### Family Orbiniidae Hartman, 1942

Falkland Islands Scratchpad http://falklands.myspecies.info/simpletaxonomy/term/250

**Phylo cf. felix Kinberg, 1866**

Falkland Islands Scratchpad http://falklands.myspecies.info/simpletaxonomy/term/255

***Phylo felix heterosetosa* Hartmann-Schröder, 1965**

Falkland Islands Scratchpad http://falklands.myspecies.info/simpletaxonomy/term/254

### Family Oweniidae Rioja, 1917

Falkland Islands Scratchpad http://falklands.myspecies.info/simpletaxonomy/term/492

***Galathowenia* sp. 1**

Falkland Islands Scratchpad http://falklands.myspecies.info/simpletaxonomy/term/492

### Family Paraonidae Cerruti, 1909

Falkland Islands Scratchpad http://falklands.myspecies.info/simpletaxonomy/term/196

**Aricidea (Acmira) cf. assimilis Tebble, 1959**

Falkland Islands Scratchpad http://falklands.myspecies.info/simpletaxonomy/term/199

**Aricidea (Acmira) simplex Day, 1963**

Falkland Islands Scratchpad http://falklands.myspecies.info/simpletaxonomy/term/203

**Aricidea (Aedicira) antarctica Hartmann-Schröder & Rosenfeldt, 1990**

Falkland Islands Scratchpad http://falklands.myspecies.info/simpletaxonomy/term/198

**Aricidea (Allia) cf. antennata Annenkova, 1934**

Falkland Islands Scratchpad http://falklands.myspecies.info/simpletaxonomy/term/200

**Aricidea (Allia) cf. ramosa Annenkova, 1934**

Falkland Islands Scratchpad http://falklands.myspecies.info/simpletaxonomy/term/201

***Aricidea pisanoi* Montiel & Hilbig, 2004**

Falkland Islands Scratchpad http://falklands.myspecies.info/simpletaxonomy/term/202

**Cirrophorus cf. furcatus Hartman, 1957**

Falkland Islands Scratchpad http://falklands.myspecies.info/simpletaxonomy/term/480

***Levinsenia acutibranchiata* Strelzov, 1973**

Falkland Islands Scratchpad http://falklands.myspecies.info/simpletaxonomy/term/205

***Levinsenia antarctica* Strelzov, 1973**

Falkland Islands Scratchpad http://falklands.myspecies.info/simpletaxonomy/term/206

***Paradoneis* sp. 1**

Falkland Islands Scratchpad http://falklands.myspecies.info/simpletaxonomy/term/207

### Family Pectinariidae Quatrefages, 1866

Falkland Islands Scratchpad http://falklands.myspecies.info/simpletaxonomy/term/468

**Pectinariidae sp. 1**

Falkland Islands Scratchpad http://falklands.myspecies.info/simpletaxonomy/term/468

### Family Pholoidae Kinberg, 1858

Falkland Islands Scratchpad http://falklands.myspecies.info/simpletaxonomy/term/469

***Pholoe* sp. 1**

Falkland Islands Scratchpad http://falklands.myspecies.info/simpletaxonomy/term/472

***Pholoe* sp. 2**

Falkland Islands Scratchpad http://falklands.myspecies.info/simpletaxonomy/term/473

### Family Phyllodocidae Örsted, 1843

Falkland Islands Scratchpad http://falklands.myspecies.info/simpletaxonomy/term/355

***Eteone* cf. aurantiaca Schmarda, 1861**

Falkland Islands Scratchpad http://falklands.myspecies.info/simpletaxonomy/term/358

**Mystides cf. notialis Ehlers, 1913**

Falkland Islands Scratchpad http://falklands.myspecies.info/simpletaxonomy/term/359

***Mystides* sp. 1**

Falkland Islands Scratchpad http://falklands.myspecies.info/simpletaxonomy/term/360

***Paranaitis* sp. 1**

Falkland Islands Scratchpad http://falklands.myspecies.info/simpletaxonomy/term/361

***Phyllodoce* sp. 1**

Falkland Islands Scratchpad http://falklands.myspecies.info/simpletaxonomy/term/363

***Phyllodoce patagonica* complex**

Falkland Islands Scratchpad http://falklands.myspecies.info/simpletaxonomy/term/362

***Protomystides* ? sp. 1**

Falkland Islands Scratchpad http://falklands.myspecies.info/simpletaxonomy/term/364

***Protomystides* ? sp. 2**

Falkland Islands Scratchpad http://falklands.myspecies.info/simpletaxonomy/term/365

### Family Pilargidae de Saint-Joseph, 1899

Falkland Islands Scratchpad http://falklands.myspecies.info/simpletaxonomy/term/259

**Ancistrosyllis cf. groenlandica**

Falkland Islands Scratchpad http://falklands.myspecies.info/simpletaxonomy/term/259

### Family Polynoidae Kinberg, 1856

Falkland Islands Scratchpad http://falklands.myspecies.info/simpletaxonomy/term/262

**Austrolaenilla cf. antarctica Bergström, 1916**

Falkland Islands Scratchpad http://falklands.myspecies.info/simpletaxonomy/term/265

***Austrolaenilla* sp. 1**

Falkland Islands Scratchpad http://falklands.myspecies.info/simpletaxonomy/term/266

***Eucranta mollis* (McIntosh, 1876)**

Falkland Islands Scratchpad http://falklands.myspecies.info/simpletaxonomy/term/267

***Euphionella patagonica* Monro, 1936**

Falkland Islands Scratchpad http://falklands.myspecies.info/simpletaxonomy/term/269

***Harmothoe magellanica* (McIntosh, 1885)**

Falkland Islands Scratchpad http://falklands.myspecies.info/simpletaxonomy/term/271

***Harmothoe anderssoni* Bergström, 1916**

Falkland Islands Scratchpad http://falklands.myspecies.info/simpletaxonomy/term/494

### Family Sabellidae Latreille, 1825

Falkland Islands Scratchpad http://falklands.myspecies.info/simpletaxonomy/term/290

***Amphicorina* sp. 2**

Falkland Islands Scratchpad http://falklands.myspecies.info/simpletaxonomy/term/314

***Amphicorina* sp. 3**

Falkland Islands Scratchpad http://falklands.myspecies.info/simpletaxonomy/term/315

***Amphicorina* sp. 4**

Falkland Islands Scratchpad http://falklands.myspecies.info/simpletaxonomy/term/316

***Amphicorina* ? sp. 1**

Falkland Islands Scratchpad http://falklands.myspecies.info/simpletaxonomy/term/313

***Desdemona* ? sp. 1**

Falkland Islands Scratchpad http://falklands.myspecies.info/simpletaxonomy/term/317

***Euchone* sp. 1**

Falkland Islands Scratchpad http://falklands.myspecies.info/simpletaxonomy/term/306

***Euchone* sp. 2**

Falkland Islands Scratchpad http://falklands.myspecies.info/simpletaxonomy/term/307

***Euchone* sp. 3**

Falkland Islands Scratchpad http://falklands.myspecies.info/simpletaxonomy/term/308

***Euchone* sp. 4**

Falkland Islands Scratchpad http://falklands.myspecies.info/simpletaxonomy/term/309

***Euchone undulocincta* Hartmann-Schröder & Rosenfeldt, 1989**

Falkland Islands Scratchpad http://falklands.myspecies.info/simpletaxonomy/term/305

***Jasmineira* (*Claviramus* ?) sp. 5**

Falkland Islands Scratchpad http://falklands.myspecies.info/simpletaxonomy/term/310

**Jasmineira cf. regularis Hartman, 1978**

Falkland Islands Scratchpad http://falklands.myspecies.info/simpletaxonomy/term/303

**Jasmineira cf. regularis (form 1)**

Falkland Islands Scratchpad http://falklands.myspecies.info/simpletaxonomy/term/302

***Jasmineira* sp. 1**

Falkland Islands Scratchpad http://falklands.myspecies.info/simpletaxonomy/term/296

***Jasmineira* sp. 2**

Falkland Islands Scratchpad http://falklands.myspecies.info/simpletaxonomy/term/297

***Jasmineira* sp. 3**

Falkland Islands Scratchpad http://falklands.myspecies.info/simpletaxonomy/term/298

***Jasmineira* sp. 4**

Falkland Islands Scratchpad http://falklands.myspecies.info/simpletaxonomy/term/299

***Jasmineira* sp. 6**

Falkland Islands Scratchpad http://falklands.myspecies.info/simpletaxonomy/term/301

**Myxicola cf. sulcata Ehlers, 1912**

Falkland Islands Scratchpad http://falklands.myspecies.info/simpletaxonomy/term/293

***Perkinsiana* sp. 1**

Falkland Islands Scratchpad http://falklands.myspecies.info/simpletaxonomy/term/320

***Perkinsiana* ? sp. 2**

Falkland Islands Scratchpad http://falklands.myspecies.info/simpletaxonomy/term/321

***Potamethus* sp. 1**

Falkland Islands Scratchpad http://falklands.myspecies.info/simpletaxonomy/term/291

### Family Scalibregmatidae Malmgren, 1867

Falkland Islands Scratchpad http://falklands.myspecies.info/simpletaxonomy/term/394

***Asclerocheilus* sp. 1**

Falkland Islands Scratchpad http://falklands.myspecies.info/simpletaxonomy/term/398

***Pseudoscalibregma bransfieldium* Hartman, 1967**

Falkland Islands Scratchpad http://falklands.myspecies.info/simpletaxonomy/term/497

***Pseudoscalibregma* sp. 1**

Falkland Islands Scratchpad http://falklands.myspecies.info/simpletaxonomy/term/399

***Scalibregma* sp. 1**

Falkland Islands Scratchpad http://falklands.myspecies.info/simpletaxonomy/term/397

***Scalibregma* sp. 2**

Falkland Islands Scratchpad http://falklands.myspecies.info/simpletaxonomy/term/400

**Scalibregmatidae sp. 1**

Falkland Islands Scratchpad http://falklands.myspecies.info/simpletaxonomy/term/396

### Family Sigalionidae Malmgren, 1867

Falkland Islands Scratchpad http://falklands.myspecies.info/simpletaxonomy/term/419

***Neoleanira magellanica* McIntosh, 1885**

Falkland Islands Scratchpad http://falklands.myspecies.info/simpletaxonomy/term/420

**Sigalionidae (*Neoleanira* ?) sp. 1**

Falkland Islands Scratchpad http://falklands.myspecies.info/simpletaxonomy/term/420

**Sigalionidae sp. 2**

Falkland Islands Scratchpad http://falklands.myspecies.info/simpletaxonomy/term/421

### Family Sphaerodoridae Malmgren, 1867

Falkland Islands Scratchpad http://falklands.myspecies.info/simpletaxonomy/term/422

***Ephesiella* ? sp. 1**

Falkland Islands Scratchpad http://falklands.myspecies.info/simpletaxonomy/term/427

**Sphaerodoropsis cf. macrotubercula Böggemann, 2009**

Falkland Islands Scratchpad http://falklands.myspecies.info/simpletaxonomy/term/423

***Sphaerodoropsis* sp. 1**

Falkland Islands Scratchpad http://falklands.myspecies.info/simpletaxonomy/term/424

***Sphaerodoropsis* sp. 2**

Falkland Islands Scratchpad http://falklands.myspecies.info/simpletaxonomy/term/425

***Sphaerodoropsis* sp. 3**

Falkland Islands Scratchpad http://falklands.myspecies.info/simpletaxonomy/term/426

***Sphaerodoropsis* sp. 4**

Falkland Islands Scratchpad http://falklands.myspecies.info/simpletaxonomy/term/498

***Sphaerodorum olgae* Moreira & Parapar, 2011**

Falkland Islands Scratchpad http://falklands.myspecies.info/simpletaxonomy/term/499

### Family Spionidae Grube, 1850

Falkland Islands Scratchpad http://falklands.myspecies.info/simpletaxonomy/term/173

***Aonidella cirrobranchiata* Day, 1961 sensu López, 2010**

Falkland Islands Scratchpad http://falklands.myspecies.info/simpletaxonomy/term/188

***Aurospio foodbancsia* Mincks et al., 2009**

Falkland Islands Scratchpad http://falklands.myspecies.info/simpletaxonomy/term/175

***Laonice* sp. 1**

Falkland Islands Scratchpad http://falklands.myspecies.info/simpletaxonomy/term/179

***Laonice* sp. 2**

Falkland Islands Scratchpad http://falklands.myspecies.info/simpletaxonomy/term/180

***Laonice* sp. 3**

Falkland Islands Scratchpad http://falklands.myspecies.info/simpletaxonomy/term/181

***Prionospio* sp.**

Falkland Islands Scratchpad http://falklands.myspecies.info/simpletaxonomy/term/185

***Prionospio* ? sp.**

Falkland Islands Scratchpad http://falklands.myspecies.info/simpletaxonomy/term/186

***Scolelepis* sp. 1**

Falkland Islands Scratchpad http://falklands.myspecies.info/simpletaxonomy/term/183

***Spiophanes algidus* Meißner, 2005**

Falkland Islands Scratchpad http://falklands.myspecies.info/simpletaxonomy/term/177

### Family Sternaspidae Carus, 1863

Falkland Islands Scratchpad http://falklands.myspecies.info/simpletaxonomy/term/256

***Sternaspis* sp. 1**

Falkland Islands Scratchpad http://falklands.myspecies.info/simpletaxonomy/term/256

### Family Syllidae Grube, 1850

Falkland Islands Scratchpad http://falklands.myspecies.info/simpletaxonomy/term/272

***Anguillosyllis palpata* Hartman, 1967**

Falkland Islands Scratchpad http://falklands.myspecies.info/simpletaxonomy/term/275

**Exogone (Paraexogone) cf. wolfi San Martín, 1991**

Falkland Islands Scratchpad http://falklands.myspecies.info/simpletaxonomy/term/280

**Exogone cf. heterosetosa McIntosh, 1885**

Falkland Islands Scratchpad http://falklands.myspecies.info/simpletaxonomy/term/277

***Exogone heterosetoides australis* Hartmann-Schröder & Rosenfeldt, 1988**

Falkland Islands Scratchpad http://falklands.myspecies.info/simpletaxonomy/term/278

***Pionosyllis* ? sp. 1**

Falkland Islands Scratchpad http://falklands.myspecies.info/simpletaxonomy/term/285

***Sphaerosyllis lateropapillata* Hartmann-Schröder, 1986**

Falkland Islands Scratchpad http://falklands.myspecies.info/simpletaxonomy/term/282

***Sphaerosyllis perspicax* Ehlers, 1912**

Falkland Islands Scratchpad http://falklands.myspecies.info/simpletaxonomy/term/500

**Syllinae sp. 1**

Falkland Islands Scratchpad http://falklands.myspecies.info/simpletaxonomy/term/501

***Syllis sclerolaema* Ehlers, 1901**

Falkland Islands Scratchpad http://falklands.myspecies.info/simpletaxonomy/term/288

***Syllis* sp. 1**

Falkland Islands Scratchpad http://falklands.myspecies.info/simpletaxonomy/term/289

### Family Terebellidae Johnston, 1846

Falkland Islands Scratchpad http://falklands.myspecies.info/simpletaxonomy/term/367

***Hauchiella* sp. 1**

Falkland Islands Scratchpad http://falklands.myspecies.info/simpletaxonomy/term/370

***Leaena* ? sp. 2**

Falkland Islands Scratchpad http://falklands.myspecies.info/simpletaxonomy/term/374

***Lysilla* sp. 1**

Falkland Islands Scratchpad http://falklands.myspecies.info/simpletaxonomy/term/371

***Pista* sp. 2**

Falkland Islands Scratchpad http://falklands.myspecies.info/simpletaxonomy/term/378

***Pista* sp. 3**

Falkland Islands Scratchpad http://falklands.myspecies.info/simpletaxonomy/term/379

***Pista* sp. 4**

Falkland Islands Scratchpad http://falklands.myspecies.info/simpletaxonomy/term/380

***Polycirrus* sp. 1**

Falkland Islands Scratchpad http://falklands.myspecies.info/simpletaxonomy/term/376

***Streblosoma* sp. 1**

Falkland Islands Scratchpad http://falklands.myspecies.info/simpletaxonomy/term/375

**Terebellinae sp. 1**

Falkland Islands Scratchpad http://falklands.myspecies.info/simpletaxonomy/term/372

### Family Travisiidae Hartmann-Schröder, 1971

Falkland Islands Scratchpad http://falklands.myspecies.info/simpletaxonomy/term/393

***Travisia* sp. 1**

Falkland Islands Scratchpad http://falklands.myspecies.info/simpletaxonomy/term/428

### Family Trichobranchidae Malmgren, 1866

Falkland Islands Scratchpad http://falklands.myspecies.info/simpletaxonomy/term/410

***Artacamella* sp. 1**

Falkland Islands Scratchpad http://falklands.myspecies.info/simpletaxonomy/term/411

***Terebellides* sp. 1**

Falkland Islands Scratchpad http://falklands.myspecies.info/simpletaxonomy/term/412

***Terebellides* sp. 2**

Falkland Islands Scratchpad http://falklands.myspecies.info/simpletaxonomy/term/413

***Terebellides* sp. 3**

Falkland Islands Scratchpad http://falklands.myspecies.info/simpletaxonomy/term/414

## Supplementary Material

XML Treatment for Harmothoe anderssoni

XML Treatment for Prosphaerosyllis modinouae

XML Treatment for Apistobranchus jasoni

XML Treatment for Leitoscoloplos olei

XML Treatment for Aphelochaeta longisetosa

XML Treatment for Aphelochaeta cf. longisetosa

XML Treatment for Aphelochaeta falklandica

XML Treatment for Dodecaceria saeria

## Supplementary materials

Supplementary material 1 Polychaete voucher specimens from East Falklands Basin, SW Atlantic accessed at NHM London Data type Biodiversity Explanation note DarwinCore database of 161 polychaete voucher specimens representing 123 morphospecies collected during 2009 and 2011 surveys from oil wells in East Falklands Basin. All voucher specimens were identified to named species or morphospecies and accessioned into collection of NHM London. https://binary.pensoft.net/file/417715This dataset is made available under the Open Database License (http://opendatacommons.org/licenses/odbl/1.0/). The Open Database License (ODbL) is a license agreement intended to allow users to freely share, modify, and use this Dataset while maintaining this same freedom for others, provided that the original source and author(s) are credited.Lenka Neal, Emma Sherlock

Supplementary material 2 Polychaete specimens (family level) from North Falklands Basin, SW Atlantic accessed at NHM London Data type Abundance, Diversity Explanation note DarwinCore database of 2 460 polychaete specimens collected during 2012 survey from Sea Lion Oil Field in North Falklands Basin. All specimens were identified to family level and accessioned into collection of NHM London. https://binary.pensoft.net/file/417716This dataset is made available under the Open Database License (http://opendatacommons.org/licenses/odbl/1.0/). The Open Database License (ODbL) is a license agreement intended to allow users to freely share, modify, and use this Dataset while maintaining this same freedom for others, provided that the original source and author(s) are credited.Lenka Neal, Emma Sherlock

Supplementary material 3 Polychaete specimens (species level) from North Falklands Basin, SW Atlantic accessed at NHM London Data type Abundance, Diversity Explanation note DarwinCore database of 15,382 polychaete specimens collected during 2012 survey from Sea Lion Oil Field in North Falklands Basin. All specimens were identified to species/morphospecies level and accessioned into collection of NHM London. https://binary.pensoft.net/file/417717This dataset is made available under the Open Database License (http://opendatacommons.org/licenses/odbl/1.0/). The Open Database License (ODbL) is a license agreement intended to allow users to freely share, modify, and use this Dataset while maintaining this same freedom for others, provided that the original source and author(s) are credited.Lenka Neal, Emma Sherlock

## References

[B1] Arntz WE (1999) Magellan-Antarctic: ecosystems that drifted apart. Summary review. Scientia Marina 63(1): 503–511. 10.3989/scimar.1999.63s1503

[B2] Averincev VG (1972) [Benthic polychaetes Errantia from the Antarctic and Subantarctic collected by the Soviet Antarctic Expedition]. Issledovaniya Fauny Morei, Zoologicheskii Institut Akademii Nauk USSR 11(19): 88–292. [In Russian]

[B3] Barnich R Fiege D Micaletto G Gambi MC (2006) Redescription of *Harmothoe spinosa* Kinberg, 1856 (Polychaeta: Polynoidae) and related species from Subantarctic and Antarctic waters, with the erection of a new genus. Journal of Natural History 40(1–2): 33–75. 10.1080/00222930500445044

[B4] Barnich R Fiege D (2009) Revision of the genus *Harmothoe* Kinberg, 1856 (Polychaeta: Polynoidae) in the Northeast Atlantic. Press, Magnolia. 10.11646/zootaxa.2104.1.1

[B5] Barnich R Fiege D (2010) On the distinction of *Harmothoe globifera* (GO. (Polychaeta, Polynoidae). Zootaxa 2525(1): 1–18. 10.11646/zootaxa.2525.1.1

[B6] Barnich R Orensanz JM Fiege D (2012) Remarks on some scale worms (Polychaeta, Polynoidae) from the Southwest Atlantic with notes on the genus *Eucranta* Malmgren, 1866, and description of a new *Harmothoe* species. Marine Biodiversity 42(3): 395–410. 10.1007/s12526-012-0117-4

[B7] Bergström E (1916) Die Polynoiden der schwedischen Südpolarexpedition 1901–1903. Zoologiska Bidrag Från Uppsala 4: 269–304.

[B8] Blake JA (1996) Family Apistobranchidae Mesnil and Caullery, 1898. In: Blake JA Hilbig B Scott PH (Eds) Taxonomic Atlas of the Benthic Fauna of the Santa Maria Basin and Western Santa Barbara Channel. 6 – The Annelida Part 3. Polychaeta: Orbiniidae to Cossuridae. Santa Barbara Museum of Natural History.Santa Barbara 6: 71–79.

[B9] Blake JA (2017) PolychaetaOrbiniidae from Antarctica, the Southern Ocean, the Abyssal Pacific Ocean, and off South America. Zootaxa 4218(1): 1–145. 10.11646/zootaxa.4218.1.1 28187682

[B10] Blake JA (2018) Bitentaculate Cirratulidae (Annelida, Polychaeta) collected chiefly during cruises of the R/V ‘Anton Bruun’, USNS Eltanin, USCG Glacier, R/V. Western South America. Zootaxa 4537(1): 1–130. 10.11646/zootaxa.4537.1.1 30647335

[B11] Blake JA Petti MAV (2019) 5.1 Apistobranchidae. In: Purschke G Böggemann M Westheide W (Eds) Handbook of Zoology.Annelida (Vol. 1): Annelida Basal groups and Pleistoannelida, Sedentaria I. De Gruyter, Berlin, 133–134. [1–480] 10.1515/9783110291582-005

[B12] Blockley D Tierney M (2017) Addressing priority gaps in understanding ecosystem functioning for the developing Falkland Islands offshore hydrocarbon industry – the ‘Gap Project’. Phase I Final Report. Environmental Forum (FIOHEF). South Atlantic Environmental Research Institute.

[B13] Bock G Fiege D Barnich R (2010) Revision of *Hermadion* Kinberg, 1856, with a redescription of *Hermadion magalhaensi* Kinberg, 1856, *Adyte hyalina* (GO Sars, 1873) n. comb. and *Neopolynoe acanellae* (Verrill, 1881) n. comb. (Polychaeta: Polynoidae). Zootaxa 2554(1): 45–61. 10.11646/zootaxa.2554.1.4

[B14] Brandt A De Broyer C Gooday AJ Hilbig B Thomson MRA (2004) Introduction to ANDEEP (ANtarctic benthic DEEP-sea biodiversity: colonization history and recent community patterns) – a tribute to Howard L. Sanders. Deep Sea Research Part II: Topical Studies in Oceanography 51: 1457–1465. 10.1016/j.dsr2.2004.08.006

[B15] Brett BJ (2001) UK Atlantic Margin Environmental Survey: introduction and overview of bathyal benthic ecology. Continental Shelf Research 8: 917–956. 10.1016/S0278-4343(00)00119-9

[B16] Carrasco FD (1977a) Polychaeta (Annelida) de Bahia de Conceptión, Chile. Familias Orbiniidae, Cirratulidae, CossuridaeCapitellidae y Ampharetidae con la descriptión de tres especies y una subspecie nuevas. Boletín de la Sociedad de Biología de Concepción 51(1): 67–92.

[B17] Carrasco FD (1977b) *Dodecaceria choromytilicola* n. sp. (Annelida, Polychaeta, Cirratulidae) perforador de *Choromytilus choros* (Mytilidae) Bolletim. Boletín de la Sociedad de Biología de Concepción 51(1): 63–66.

[B18] Dahlgren TG Wiklund H Rabone M Amon DJ Ikebe C Watling L Smith CR Glover AG (2016) Abyssal fauna of the UK-1 polymetallic nodule exploration area, Clarion-Clipperton Zone, central Pacific Ocean: Cnidaria Biodiversity Data Journal 4. 10.3897/BDJ.4.e9277 PMC5018120 27660533

[B19] De Nogueira JM San Martín G Amaral AC (2001) Description of five new species of Exogoninae Rioja, 1925 (Polychaeta: Syllidae) associated with the stony coral *Mussismilia hispida* (Verrill, 1868) in Sao Paulo State, Brazil. Journal of Natural History 35(12): 1773–1794. 10.1080/00222930152667096

[B20] Ehlers E (1901) Die Polychaeten des magellanischen und chilenischen Strandes. Ein faunistischer Versuch. Festschrift zur Feier des Hundertfünfzigjährigen Bestehens des Königlichen Gesellschaft der Wissenschaften zu Göttingen, Abhandlungen der Mathematisch-Physikalischen Klasse, 232 pp.

[B21] Elias R Rivero MS (2009a) First new *Dodecaceria* (Polychaeta: Cirratulidae) species from the SW Atlantic (38°S -57°W, Argentina). Revista de Biologia Marina y Oceanografia 44: 131–136. 10.4067/S0718-19572009000100012

[B22] Elias R Rivero MS (2009b) Two new species of Cirratulidae (Annelida: Polychaeta) from Mar del Plata. Argentina (SW Atlantic). Zoosymposia 2: 139–148. 10.1590/S0073-47212008000200010

[B23] Fauvel P (1950) Missions du batiment polaire “Commandant-Charcot” récoltes faites en Terre Adélie (1950) I. Annélides Polychètes. Bulletin du Muséum d’Histoire Naturelle, Paris, Series 2 22(6): 753–773.

[B24] Fukuda MV Yunda-Guarin G Nogueira JM (2009) The genus *Prosphaerosyllis* (Polychaeta: Syllidae: Exogoninae) in Brazil, with description of a new species. Journal of the Marine Biological Association of the United Kingdom 89(7): 1443–1454. 10.1017/S0025315409000095

[B25] Glover AG Dahlgren TG Wiklund H Mohrbeck I Smith CR (2015) An end-to-end DNA taxonomy methodology for benthic biodiversity survey in the Clarion-Clipperton Zone, central Pacific abyss. Journal of Marine Scienceand Engineering 4(1): 1–2. 10.3390/jmse4010002

[B26] Hartmann-Schröder G (1962) Zur Kenntnis des Eulitorals der chilenischen Pazifikküste und der argentinischen Küste Südpatagoniens unter besonderer Berücksichtigung der Polychaeten und Ostracoden (Vol. 60). Zoologisches Staatsinstitut und Zoologisches Museum.

[B27] Hartmann-Schröder G (1965) Zur Kenntnis des Sublitorals der chilenischen Küste unter besonderer Berücksichtigung der Polychaeten und Ostracoden: mit Bemerkungen über den Einfluß sauerstoffarmer Strömungen auf die Besiedlung von marinen Sedimenten. Zoologisches Staatsinstitut und Zoologisches Museum.

[B28] Hartmann-Schröder G Rosenfeldt P (1988) The polychaetes of the Ant III-2 voyage of the Polarstern to the Antarctic 1984. 85. Mitteilungen aus dem Hamburgischen Zoologischen Museum und Institut, 25–72.

[B29] Hartmann-Schröder G Rosendfeldt P (1989) Zoogeography and ecology of the polychaetes from ANT III/2 of R/V ‘Polarstern’to Antarctica 1984. Third International Polychaete held at California State University, Long Beach California.

[B30] Hartmann-Schröder G Rosenfeldt P (1990) Die Polychaeten der “Walther Herwig”-Reise 68/1 nach Elephant Island (Antarktis). Mitteilungen aus den Hamburgischen Zoologischen Museum und Institut 87: 89–122.

[B31] Hartmann-Schröder G Rosenfeldt P (1992) Die Polychaeten der “Polarstern” -Reise ANT V/1 in die Antarktis 1986. Teil 1: Euphrosinidae bis Iphitimidae. Mitteilungen aus den Hamburgischen Zoologischen Museum und Institut 89: 85–124.

[B32] Hartman O (1953) Non-pelagic polychaeta of the Swedish Antarctic Expedition 1901–1903. Further Zoological Results of the Swedish Antarctic Expedition 1901–1903 4(11): 1–83.

[B33] Hartman O (1965) Deep-water benthic polychaetous annelids off New England to Bermuda and other North Atlantic areas. Occasional Papers of the Allan Hancock Foundation 28: 1–378.

[B34] Hartman O (1966) PolychaetaMyzostomidae and Sedentaria of Antarctica. Antarctic Research Series 7: 1–158. 10.1029/AR007

[B35] Hartman O (1967) Polychaetous annelids collected by the USNS Eltanin and Staten Island cruises, chiefly from Antarctic seas. (No. 2). Allan Hancock Foundation, University of Southern California, 1–387.

[B36] Hartman O (1978) Polychaeta from the Weddell Sea Quadrant. Antarctica. American Geophysical Union, Washington, 125–223. 10.1029/AR026p0125

[B37] Hedgpeth JW (1969) Introduction to Antarctic zoogeography. Distribution of selected groups of marine invertebrates in waters south of 35°S latitude. Antarctic Map Folio Series 11: 1–9.

[B38] HeliconSoft (2018) Helicon Focus. HeliconSoft. https://www.heliconsoft.com

[B39] Kaiser S Barnes DK Sands CJ Brandt A (2009) Biodiversity of an unknown Antarctic Sea: assessing isopod richness and abundance in the first benthic survey of the Amundsen continental shelf. Marine Biodiversity 39(1): 1–27. 10.1007/s12526-009-0004-9

[B40] Mackie AS (1987) A review of species currently assigned to the genus *Leitoscoloplos* Day. Sarsia 72(1): 1–28. 10.1080/00364827.1987.10419701

[B41] Miranda VD Brasil AC (2014) Two new species and a new record of scale-worms (Polychaeta) from Southwest Atlantic deep-sea coral mounds. Zootaxa 3856(2): 1–211. 10.11646/zootaxa.3856.2.3 25284654

[B42] Monro CC (1930) Polychaete worms. Discovery Reports, Cambridge 2: 1–222.

[B43] Monro CC (1936) Polychaete worms II. Discovery Reports, Cambridge 12: 59–198.

[B44] Moreau C Linse K Griffiths H Barnes D Kaiser S Glover A Sands C Strugnell J Enderlein P Geissler P (2013) Amundsen Sea Mollusca from the BIOPEARL II expedition. ZooKeys 294: 1–8. 10.3897/zookeys.294.4796 PMC3677319 23794869

[B45] Neal L Barnich R Wiklund H Glover AG (2012) A new genus and species of Polynoidae (Annelida, Polychaeta) from Pine Island bay, Amundsen Sea, Southern Ocean-a region of high taxonomic novelty. Zootaxa 3542: 80–88. 10.11646/zootaxa.3542.1.4

[B46] Neal L Linse K Brasier MJ Sherlock E Glover AG (2017) Comparative marine biodiversity and depth zonation in the Southern Ocean: evidence from a new large polychaete dataset from Scotia and Amundsen seas. Marine Biodiversity 48: 581–601. 10.1007/s12526-017-0735-y

[B47] Norlinder E Nygren A Wiklund H Pleijel F (2012) Phylogeny of scale-worms (Aphroditiformia, Annelida), assessed from 18SrRNA, 28SrRNA, 16SrRNA, mitochondrial cytochrome c oxidase subunit I (COI), and morphology. Molecular Phylogenetics and Evolution 65(2): 490–500. 10.1016/j.ympev.2012.07.002 22789762

[B48] Nygren A (2014) Cryptic polychaete diversity: a review. Zoologica Scripta 43(2): 172–183. 10.1111/zsc.12044

[B49] Pabis K Błażewicz-Paszkowycz M Jóźwiak P Barnes DK (2015) Tanaidacea of the Amundsen andScotia seas: an unexplored diversity. Antarctic Science 1: 19–30. 10.1017/S0954102014000303

[B50] Petti MA Nonato EF Bromberg S Gheller PF Paiva PC Corbisier TN (2007) On the taxonomy of *Apistobranchus* species (Polychaeta: Apistobranchidae) from the Antarctic. Zootaxa 1440: 51–59. 10.11646/zootaxa.1440.1.4

[B51] Rozbaczylo N (1985) Los Anélidos Poliquetos de Chile. Monografias Biológicas 3: 1–284.

[B52] San Martín G (1984) Descripción de una nueva especie y revisión del género *Sphaerosyllis* (Polychaeta: Syllidae). Cahiers de Biologie Marine 25(4): 375–391.

[B53] San Martín G (2005) Exogoninae (Polychaeta: Syllidae) from Australia with the description of a new genus and twenty-two new species. Records of the Australian Museum 57(1): 39–152. 10.3853/j.0067-1975.57.2005.1438

[B54] Schüller M Ebbe B Wägele JW (2009) Community structure and diversity of polychaetes (Annelida) in the deep Weddell Sea (Southern Ocean) and adjacent basins. Marine Biodiversity 39(2): 95–108. 10.1007/s12526-009-0009-4

[B55] Schüller M Jirkov IA (2013) New Ampharetidae (Polychaeta) from the deep Southern Ocean and shallow Patagonian waters. Zootaxa 3692(1): 204–237. 10.11646/zootaxa.3692.1.11

[B56] Smith VS Rycroft S Scott B Baker E Livermore L Heaton A Boulton K Koureas DN Roberts D (2012) Scratchpads 2.0: a virtual research environment infrastructure for biodiversity data. http://scratchpads.eu

[B57] Webster H Benedict EJ (1887) The AnnelidaChaetopoda, from Eastport, Maine. U.S. Commission of Fish & Fisheries. Report of the United States Commissioner of Fisheries. 1885 appendix to report of commissioner, D.22, Eastport, 707–758.

